# Association between aphasia severity and brain network alterations after stroke assessed using the electroencephalographic phase synchrony index

**DOI:** 10.1038/s41598-021-91978-7

**Published:** 2021-06-14

**Authors:** Teiji Kawano, Noriaki Hattori, Yutaka Uno, Megumi Hatakenaka, Hajime Yagura, Hiroaki Fujimoto, Michiko Nagasako, Hideki Mochizuki, Keiichi Kitajo, Ichiro Miyai

**Affiliations:** 1 Neurorehabilitation Research Institute, Morinomiya Hospital, Osaka, 536-0025 Japan; 2 Department of Neurology, Graduate School of Medicine, Osaka University, Osaka, 565-0871 Japan; 3 Rhythm-Based Brain Information Processing Unit, RIKEN CBS-TOYOTA Collaboration Center, RIKEN Center for Brain Science, Wako, 351-0198 Japan; 4 Department of Rehabilitation, Faculty of Medicine, Academic Assembly, University of Toyama, Toyama, 930-0194 Japan; 5 Division of Neural Dynamics, Department of System Neuroscience, National Institute for Physiological Sciences, National Institutes of Natural Sciences, Okazaki, 444-8585 Japan; 6 Department of Physiological Sciences, School of Life Science, The Graduate University for Advanced Studies (SOKENDAI), Okazaki, 444-8585 Japan

**Keywords:** Diseases of the nervous system, Regeneration and repair in the nervous system, Stroke, Disability, Language

## Abstract

Electroencephalographic synchrony can help assess brain network status; however, its usefulness has not yet been fully proven. We developed a clinically feasible method that combines the phase synchrony index (PSI) with resting-state 19-channel electroencephalography (EEG) to evaluate post-stroke motor impairment. In this study, we investigated whether our method could be applied to aphasia, a common post-stroke cognitive impairment. This study included 31 patients with subacute aphasia and 24 healthy controls. We assessed the expressive function of patients and calculated the PSIs of three motor language-related regions: frontofrontal, left frontotemporal, and right frontotemporal. Then, we evaluated post-stroke network alterations by comparing PSIs of the patients and controls and by analyzing the correlations between PSIs and aphasia scores. The frontofrontal PSI (beta band) was lower in patients than in controls and positively correlated with aphasia scores, whereas the right frontotemporal PSI (delta band) was higher in patients than in controls and negatively correlated with aphasia scores. Evaluation of artifacts suggests that this association is attributed to true synchrony rather than spurious synchrony. These findings suggest that post-stroke aphasia is associated with alternations of two different networks and point to the usefulness of EEG PSI in understanding the pathophysiology of aphasia.

## Introduction

Aphasia, a common post-stroke manifestation affecting 15‒42% of patients^1^, often leads to a poor functional outcome^2^. Thus, to understand the pathophysiological underpinnings of aphasia, great efforts have been made to establish clinically applicable tools that can assess damaged brain function^3^.

Since language function is lateralized to the dominant hemisphere, many studies have focused on stroke lesions in the left (ipsilesional) hemisphere^4^. In addition, recent reports with functional imaging data have indicated that functional connectivity (FC) can offer new insights into changes remote from the stroke lesion, including the right (contralesional) homologous brain area^5^. Magnetic resonance imaging (MRI) is the most commonly used modality in FC studies. A large cohort resting-state functional MRI (fMRI) study revealed that both lesion topography and FC were equally useful for explaining aphasia severity and that decreased interhemispheric FC of the language-related regions correlated with aphasia scores^6^. Electroencephalographic synchrony analysis can also be performed to assess neural network functions, because FC is defined as the temporal correlation of physiological signals in remote brain regions^7^. Furthermore, electroencephalography (EEG) can offer a high temporal resolution, providing distinct information in various frequency bands, and allowing noninvasive recording at a low cost^8^. A combination of high-density EEG (128-channel) and coherence analysis with a graph-theoretical approach was reported to predict recovery from post-stroke aphasia^9^.

However, fMRI and high-density EEG recordings cannot be performed in most medical facilities. Practically, a more clinically feasible recording approach would be preferable. Recently, we have reported that the phase synchrony index (PSI), computed from resting-state 19-channel EEG, reflects post-stroke brain network status and is associated with motor impairment and recovery in patients with hemiparesis^10^. Theoretically, the PSI is robust to amplitude change and selectively indicates EEG synchrony^11,12^. Specifically, the PSI between a pair of electrodes on the bilateral primary motor cortices decreased in subacute stroke and positively correlated with the upper extremity Fugl–Meyer motor assessment score.

**Figure 1:**
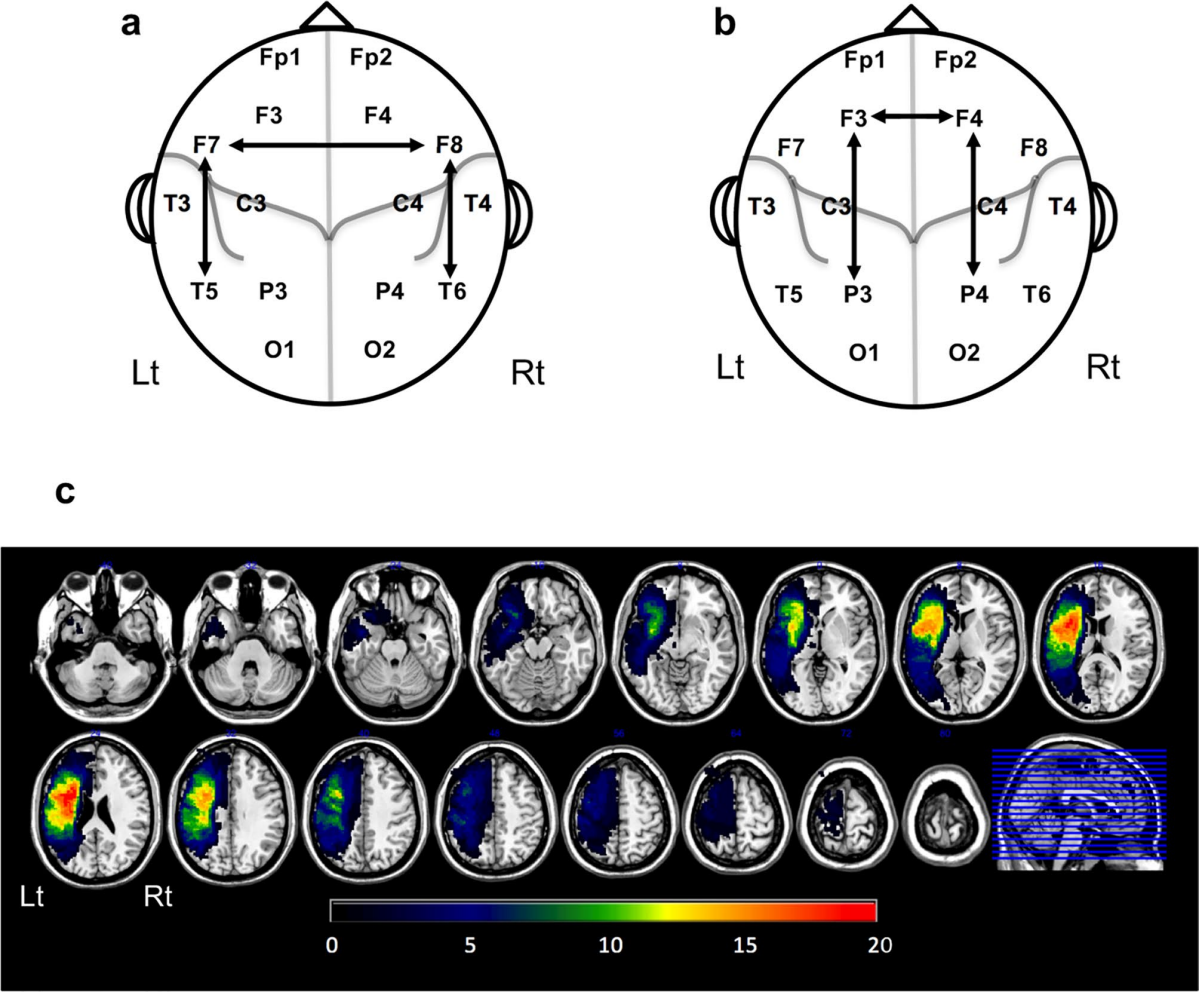
Maps of electrode pairs and stroke lesions in this study. (**a**) Electrode pairs used for the computation of the F7F8-PSI, F7T5-PSI, and F8T6-PSI. They are located in the motor language-related regions. (**b**) Electrode pairs (F3F4, F3P3, and F4P4) spatially adjacent to those in (**a**) (F7F8, F7T5, and F8T6) used for evaluation of the effect of artifacts. They are not located in the motor language-related regions. (**c**) A map of the ischemic stroke lesion distribution in 31 patients. The colors indicate the number of patients with lesions. *PSI* phase synchrony index.

In the current cross-sectional study, we sought to test if this PSI method could be applied to the assessment of aphasia, one of the most common post-stroke cognitive impairments. In our study, the choice of electrodes was based on existing neuroanatomical^13–16^ and neurophysiological^6,17,18^ findings regarding aphasia. We hypothesized that the PSIs between the motor language-related regions would reflect post-stroke brain network alterations and would correlate with the expressive function of patients with aphasia. Specifically, we concentrated on the left inferior frontal lobe (Broca’s area) and its right hemispheric homotopic region, considering their anatomical connection via the corpus callosum. We also focused on the frontotemporal networks of long association fibers connecting Broca’s and Wernicke’s areas. In addition, we focused on right homotopic frontotemporal network. Based on the 19-channel EEG electrode setting, we focused on three PSIs related to the left (F7) and right (F8) inferior frontal lobes: (1) the interhemispheric frontofrontal (F7F8) PSI, (2) left intrahemispheric frontotemporal (F7T5) PSI, and (3) right intrahemispheric frontotemporal (F8T6) PSI (Fig. 1a). First, we assessed post-stroke network alterations by comparing PSIs of patients with those of healthy controls. Then, we evaluated correlations of these PSIs with the Aphasia Rating Scale speech (ARSsp) score^19^ calculated from seven subscores of the Standard Language Test of Aphasia (SLTA)^20^. Furthermore, we evaluated the possibility of spurious synchrony due to artifacts^21^ by assessing the spatially adjacent electrode pairs that are not located in the motor language-related regions (Fig. 1b), the global PSIs, the effect of stroke lesion volume (LV), and another synchrony measure: Phase Lag Index (PLI). The PLI may underestimate true synchrony due to its conservative nature^22^. In addition, we also evaluated the EEG power to compare with the PSI.

## Results

### Participants’ characteristics

This study included 31 subacute stroke patients (mean age: 67.6 years, seven women) with aphasia and 24 age- and sex-matched healthy controls. All patients had ischemic cortical lesions in the left frontal lobe (Fig. 1c). We recorded the resting-state EEG (median: 35.0 days after stroke). The median interval between EEG recording and the SLTA assessment was 2.0 days (interquartile range: − 1.0 to 5.0 days). Table 1 summarizes the demographic and clinical characteristics of the participants. The Shapiro–Wilk test revealed that the ARSsp scores, LV, and substantial proportions of the PSIs and the PLIs deviated from a normal distribution. Detailed data of each patient and each healthy control participant are described in the Supplementary information (Supplementary Tables S1‒S30 online).

**Table 1:** Demographic and clinical characteristics of the participants.

Variables	Patients	Healthy controls	Statistics	*P*
Number of participants	31	24	–	–
Age, (years, ± SD)	67.6 ± 13.2	66.0 ± 6.9	*t*_(47.3)_ = − 0.73	0.471
Women:Men	7:24	10 : 14	*χ*^*2*^_(1)_ = 0.52	0.472
MMSE score (median [IQR])	–	30.0 [28.5–30.0]	–	–
EEG recording after stroke onset (days, median [IQR])	35.0 [27.8–43.0]	–	–	–
NIHSS score (median [IQR])	6 [2–10]	–	–	–
ARSsp score (median [IQR])	47.0 [3.5–62.0]	–	–	–
LV (mm^3^; median [IQR])	59,704 [33,464–103,216]	–	–	–

*ARSsp* Aphasia Rating Scale speech, *EEG* electroencephalography, *IQR* interquartile range, *LV* lesion volume, *MMSE* mini mental state examination, *NIHSS* National Institutes of Health Stroke Scale, *SD* standard deviation.

### Evaluation of the PSIs between motor language-related regions

**Figure 2:**
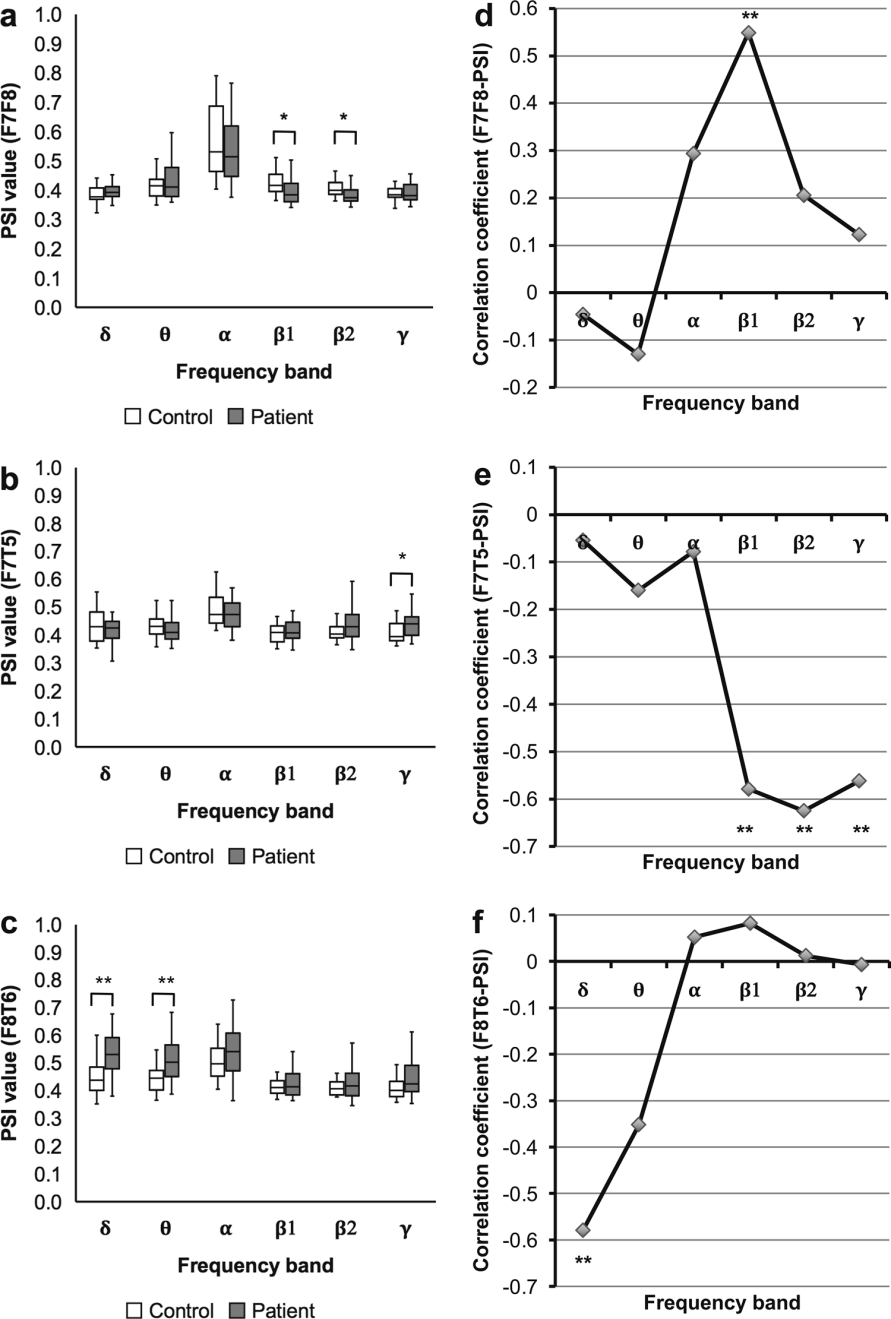
Results of analyses for the PSIs between the motor language-related regions. Comparison of PSIs in stroke patients with those in healthy controls. A box-and-whisker plot indicating values of the (**a**) F7F8-PSI, (**b**) F7T5-PSI, and (**c**) F8T6-PSI of stroke patients and healthy controls (Mann‒Whitney U test; * *P* < 0.05; ** *P* < 0.01, FDR corrected). The F7F8-PSI (β1 and β2), the F7T5-PSI (γ), and the F8T6-PSI (δ and θ) show significant differences between stroke patients and healthy controls. Correlation coefficients of the F7F8/F7T5/F8T6-PSIs with ARSsp scores. A polygonal line graph of the correlation coefficients for the correlation of (**d**) F7F8-PSI, (**e**) F7T5-PSI, and (**f**) F8T6-PSI with ARSsp scores (Spearman’s correlation analysis; * *P* < 0.05; ** *P* < 0.01, FDR corrected). Each PSI shows significant correlations in distinct frequency bands with significant differences between stroke patients and healthy controls. *ARSsp* Aphasia Rating Scale speech, *FDR* false discovery rate, *PSI* phase synchrony index.

First, we evaluated three PSIs between motor language-related regions according to our hypothesis. We assessed post-stroke network alterations by comparing PSIs of patients with those of healthy controls. The F7F8-PSI in patients was significantly lower than that in healthy controls in the β1 and β2 bands (β1: *U* = 213.0, *P* = 0.031; β2: *U* = 193.0, *P* = 0.014). The F7T5-PSI in patients was significantly higher than that in healthy controls in the γ band (*U* = 221.0, *P* = 0.037). The F8T6-PSI in patients was significantly higher than that in healthy controls in the δ and θ bands (δ: *U* = 149.0, *P* = 0.003; θ: *U* = 164.0, *P* = 0.004; Fig. 2a‒c; Supplementary Table S31 online). Results are false discovery rate (FDR) corrected (*P* < 0.05).

**Figure 3:**
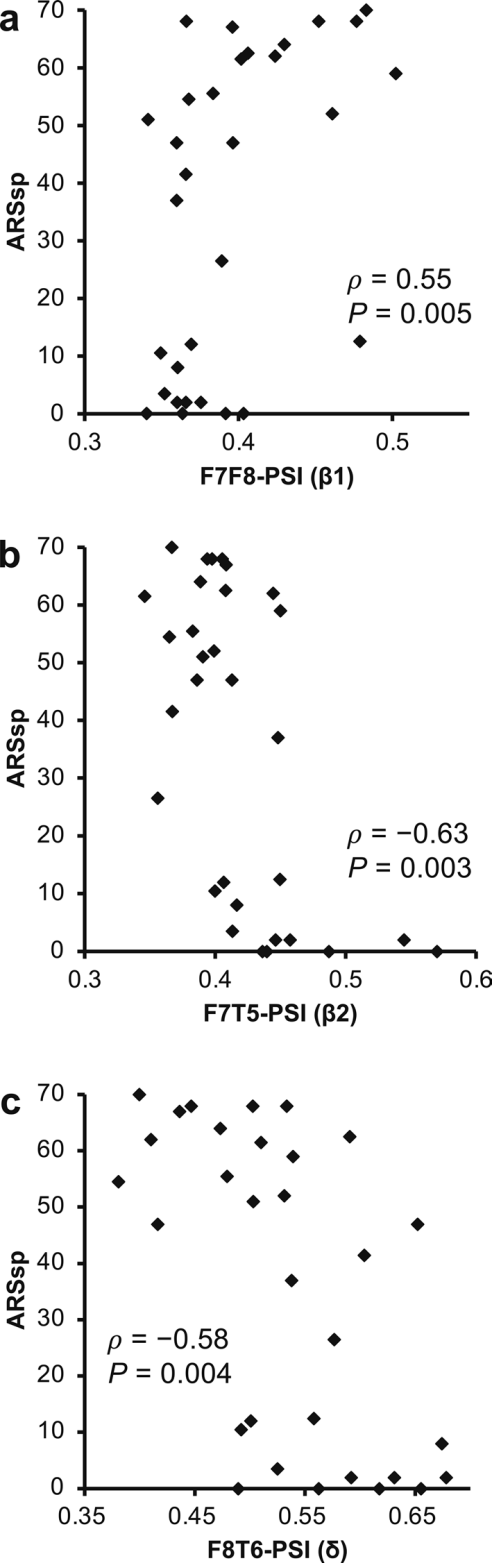
Correlation of the F7F8/F7T5/F8T6-PSIs with ARSsp scores. Scatter plots of the (**a**) F7F8-PSI, (**b**) F7T5-PSI, and (**c**) F8T6-PSI versus the ARSsp scores (*P* values: Spearman’s correlation analysis; FDR corrected). The F7F8-PSI is correlated positively with the ARSsp score, whereas the F7T5-PSI and F8T6-PSI are correlated negatively with the ARSsp scores. *ARSsp* Aphasia Rating Scale speech, *FDR* false discovery rate, *PSI* phase synchrony index.

Next, we performed a correlation analysis between the F7F8/F7T5/F8T6-PSIs and ARSsp scores. The F7F8-PSI correlated significantly positively with the ARSsp score in the β1 band (*ρ* = 0.55, *P* = 0.005). By contrast, the F7T5-PSI was significantly negatively correlated with the ARSsp scores in the β1, β2, and γ bands (β1: *ρ* =  − 0.58, *P* = 0.006; β2: *ρ* =  − 0.63, *P* = 0.003; γ: *ρ* =  − 0.56, *P* = 0.005). The largest correlation coefficient was observed for the β2 band. On the other hand, the F8T6-PSI was significantly negatively correlated with the ARSsp score in the δ band (*ρ* =  − 0.58, *P* = 0.004; Fig. 2d‒f; *P* < 0.05, FDR corrected; Supplementary Table S32 online). Scatter plots (Fig. 3a‒c) revealed a positive correlation of the F7F8-PSI (β1) with the ARSsp score and negative correlations between the F7F5-PSI (β2)/F8T6-PSIs (δ) and ARSsp scores.

### Evaluation of the PSIs between adjacent electrode pairs

**Figure 4:**
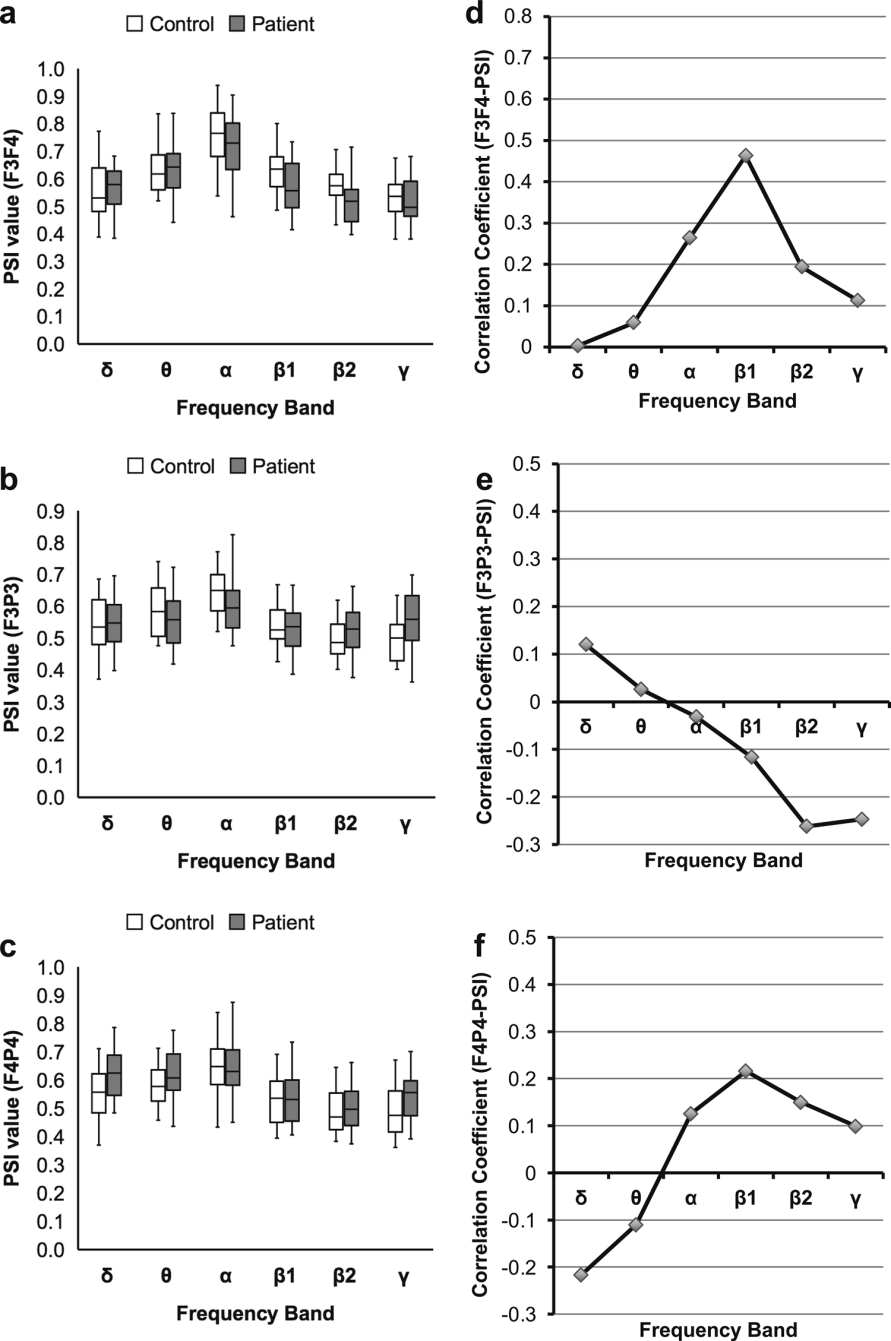
Results of analyses for the spatially adjacent PSIs. Comparison of PSIs in stroke patients with those in healthy controls. A box-and-whisker plot indicating values of the (**a**) F3F4-PSI, (**b**) F3P3-PSI, and (**c**) F4P4-PSI of stroke patients and healthy controls (Mann‒Whitney U test, FDR corrected). In contrast to the PSIs between motor language-related regions, all spatially adjacent PSIs show no significant difference between stroke patients and healthy controls. Correlation coefficients of the F3F4/F3P3/F4P4-PSIs with ARSsp scores. A polygonal line graph of the correlation coefficients for the correlation of (**d**) F3F4-PSI, (**e**) F3P3-PSI, and (**f**) F4P4-PSI with ARSsp scores (Spearman’s correlation analysis, FDR corrected). In contrast to the PSIs between motor language-related regions, all spatially adjacent PSIs show no significant correlations in any frequency band. *ARSsp* Aphasia Rating Scale speech, *FDR* false discovery rate, *PSI* phase synchrony index.

Second, to evaluate the possibility of spurious synchrony due to artifacts, we assessed spatially adjacent electrode pairs (F3F4/F3P3/F4P4; Fig. 1b) that are not located in the motor language-related regions. We found that the F3F4/F3P3/F4P4-PSIs in patients showed no significant difference from those in healthy controls (*P* < 0.05, FDR corrected; Fig. 4a‒c; Supplementary Table S31 online).

We then performed correlation analysis between the F3F4/F3P3/F4P4-PSIs and ARSsp scores. We found that the F3F4/F3P3/F4P4-PSIs showed no significant correlation with ARSsp scores in any frequency band (*P* < 0.05, FDR corrected; Fig. 4d‒f; Supplementary Table S32 online).

### Evaluation of the global PSI

**Figure 5:**
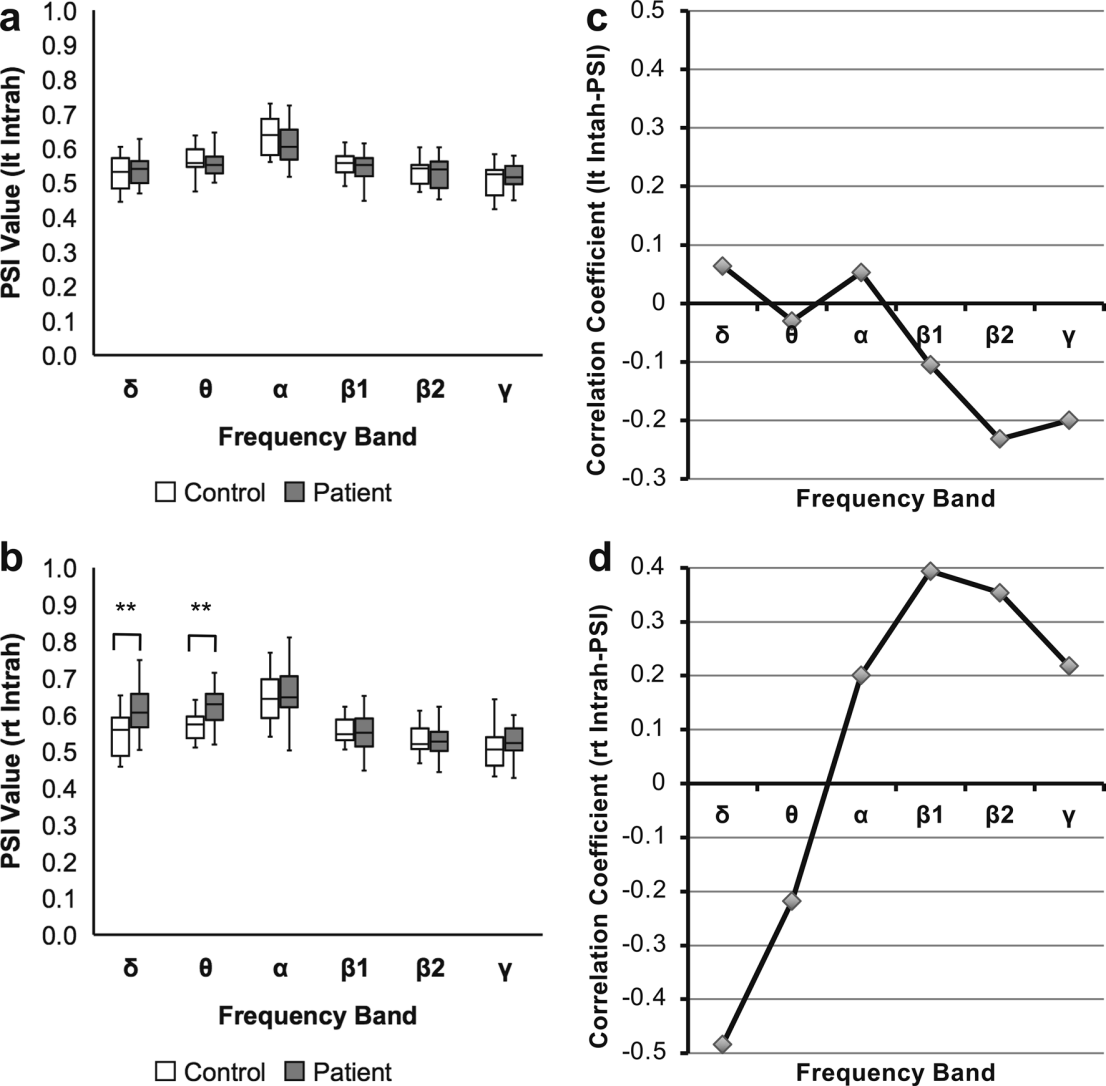
Results of analyses for the global PSIs. Comparison of PSIs in stroke patients with those in healthy controls. A box-and-whisker plot indicating values of the (**a**) left Intrah-PSI and (**b**) right Intrah-PSI of stroke patients and healthy controls (Mann‒Whitney U test; * *P* < 0.05, ** *P* < 0.01, FDR corrected). Right Intrah-PSIs shows significant differences between stroke patients and healthy controls in the δ and θ bands. Correlation coefficients of the left/right Intrah-PSIs with ARSsp scores. A polygonal line graph of the correlation coefficients for the correlation of (**c**) left Intrah-PSI and (**d**) right Intrah-PSI with ARSsp scores (Spearman’s correlation analysis, FDR corrected). In contrast to the PSIs between motor language-related regions, global PSIs show no significant correlations in any frequency band. *ARSsp* Aphasia Rating Scale speech, *FDR* false discovery rate, *Intrah* intrahemispheric, *lt* left, *PSI* phase synchrony index, *rt* right.

Third, to compare with the local synchrony of motor language-related regions, we evaluated the global intrahemispheric PSI (Intrah-PSI) of both hemispheres. The left Intrah-PSI in patients showed no significant difference from that in healthy controls, while right Intrah-PSI in patients was significantly higher than that in healthy controls in the δ and θ bands (Fig. 5a and b; δ: *U* = 161.0, *P* = 0.004; θ: *U* = 165.0, *P* = 0.003; *P* < 0.05, FDR corrected; Supplementary Table S33 online). In the correlation analysis, however, we found that either the left or right Intrah-PSIs showed no significant correlation with ARSsp scores in any frequency band (*P* < 0.05, FDR corrected; Fig. 5c and d; Supplementary Table S34 online).

### Evaluation of the PLIs between motor language-related regions

**Figure 6:**
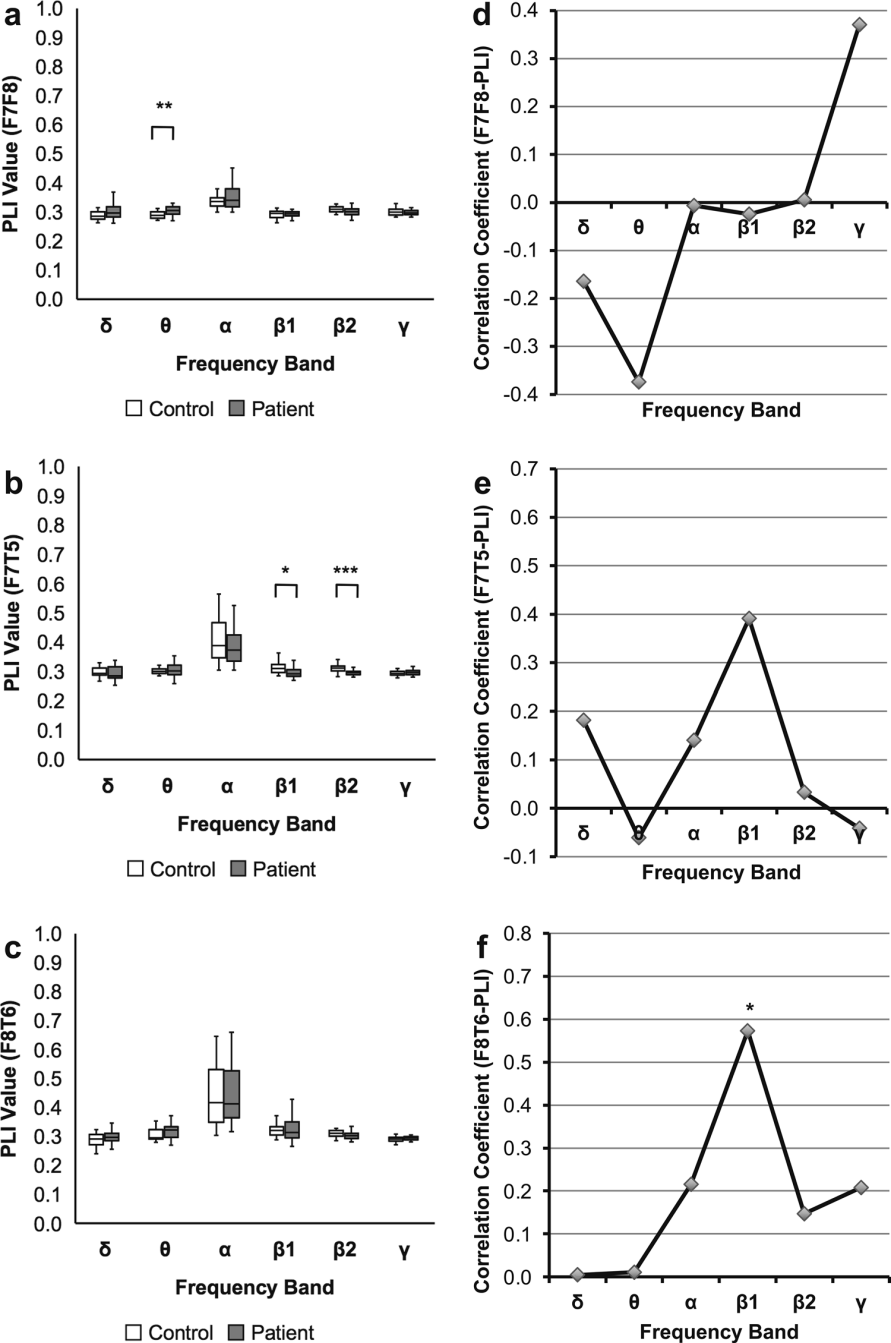
Results of analyses for the PLIs between the motor language-related regions. Comparison of the PLIs in stroke patients with those in healthy controls. A box-and-whisker plot indicating values of the (**a**) F7F8-PLI, (**b**) F7T5-PLI, and (**c**) F8T6-PLI of stroke patients and healthy controls (Mann‒Whitney U test; * *P* < 0.05, ** *P* < 0.01, *** *P* < 0.001, FDR corrected). In contrast to the F7F8-PLI and F7T5-PLI, the F8T6-PLI shows no significant difference between stroke patients and healthy controls. Correlation coefficients of the F7F8/F7T5/F8T6-PLIs with ARSsp scores. A polygonal line graph of the correlation coefficients for the correlation of (d) F7F8-PLI, (e) F7T5-PLI, and (f) F8T6-PLI with ARSsp scores (Spearman’s correlation analysis; * *P* < 0.05, FDR corrected). In contrast to the F7F8-PLI and F7T5-PLI, the F8T6-PLI shows a significant correlation in the β1 band. *ARSsp* Aphasia Rating Scale speech, *FDR* false discovery rate, *PLI* phase lag index.

Fourth, for further evaluation of the effect due to artifacts, we assessed the F7F8/F7T5/F8T6-PLIs because the PLI is less affected by the artifacts originating from near-zero phase synchrony^22^ compared with the PSI. The F7F8-PLI in patients was significantly higher than that in healthy controls in the θ band (*U* = 160.0, *P* = 0.003). The F7T5-PLI in patients was significantly lower than that in healthy controls in the β1 and β2 bands (β1: *U* = 200.0, *P* = 0.024; β2: *U* = 124.0, *P* < 0.001). The F8T6-PLI in patients showed no significant difference from that in healthy controls (*P* < 0.05, FDR corrected; Fig. 6a‒c; Supplementary Table S35 online).

We next performed correlation analysis between the F7F8/F7T5/F8T6-PLIs and ARSsp. We found that the F7F8-PLI and F7T5-PLI were not correlated with the ARSsp scores, while F8T6-PLI was significantly positively correlated with the ARSsp score in the β1 band (*ρ* = 0.57, *P* = 0.013; *P* < 0.05, FDR corrected; Fig. 6d‒f; Supplementary Table S36 online).

### Evaluation of EEG power of motor language-related regions

Fifth, for comparison with the PSI, we assessed the EEG wavelet power (wP) related to F7F8, F7T5, and F8T6. The F7F8-wP in patients showed no significant difference from that in healthy controls. The F7T5-wP in patients was significantly higher than that in healthy controls in the β1 band (β1: *U* = 204.0, *P* = 0.039). The F8T6-wP in patients was significantly higher than that in healthy controls in the β2 band (*U* = 209.0, *P* = 0.034; *P* < 0.05, FDR corrected; Supplementary Table S37 online). In the correlation analysis, however, the F7F8/F7T5/F8T6-wP showed no significant correlation with the ARSsp scores in any frequency band (*P* < 0.05, FDR corrected; Supplementary Table S38 online).

### Evaluation of the stroke LV

Finally, we assessed the effect of stroke LV on the synchrony analysis. The F7T5-PSI was significantly negatively correlated with LV in the β1, β2, and γ bands (β1: *ρ* = 0.56, *P* = 0.010; β2: *ρ* = 0.62, *P* = 0.003; γ: *ρ* = 0.55, *P* = 0.009; *P* < 0.05, FDR corrected). In contrast, the F7F8/F8T6-PSIs and the F7F8/F7T5/F8T6-PLIs showed no significant correlation with LV in any frequency band (*P* < 0.05, FDR corrected; Supplementary Table S39‒S40 online).

**Figure 7:**
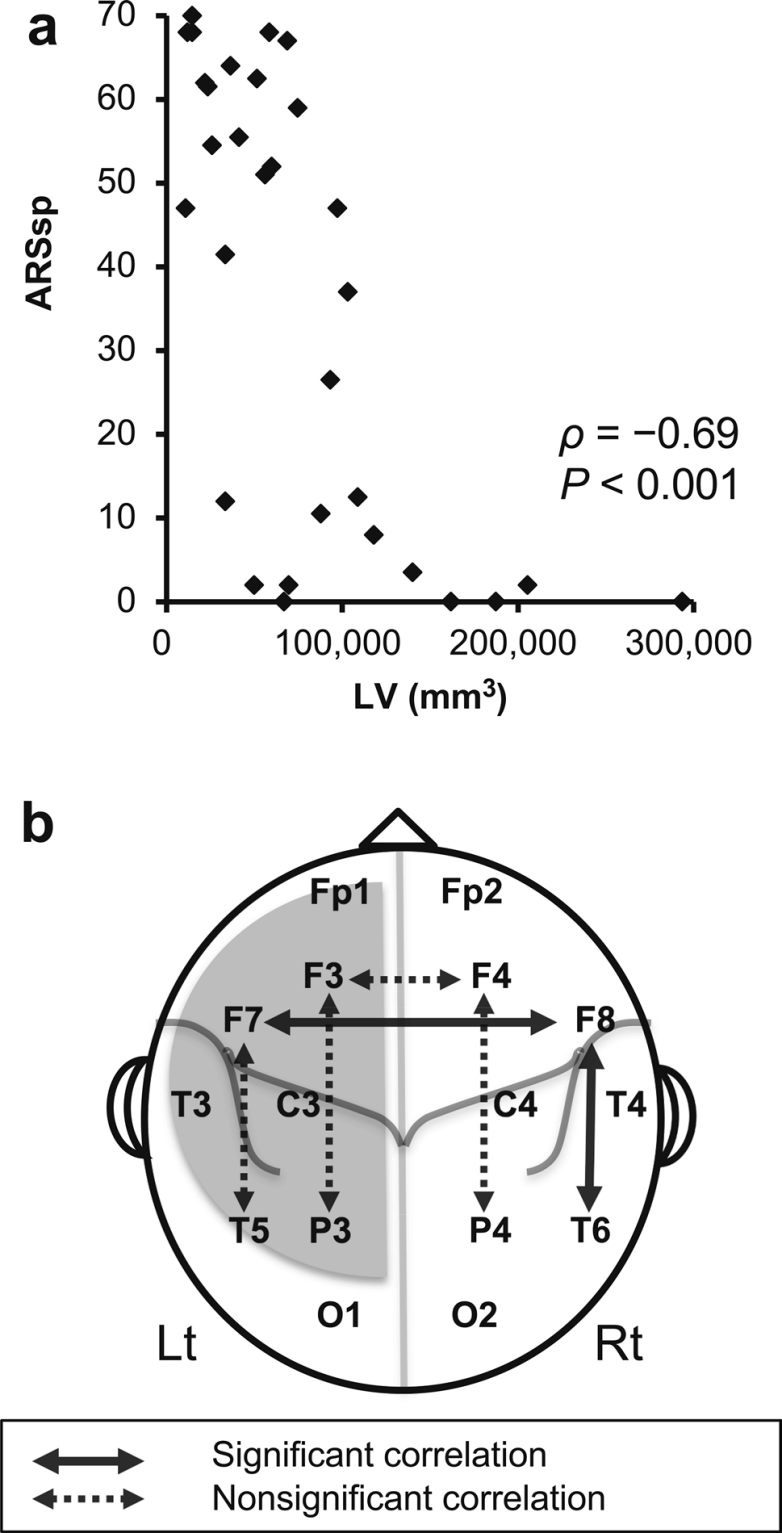
A correlation of LV with ARSsp scores and a summary of correlation analyses in this study. (**a**) A scatter plot showing a negative correlation between stroke LV and the ARSsp score. (**b**) A summary of the correlation analyses between six PSIs (F7F8-PSI, F3F4-PSI, F7T5-PSI, F3P3-PSI, F8T6-PSI, and F4P4-PSI)/the F8T6-PLI and ARSsp scores is plotted on the brain overlay. The solid arrow indicates a significant correlation, whereas dashed arrows indicate nonsignificant correlations. The gray shadow indicates the stroke lesion. *ARSsp* Aphasia Rating Scale speech, *LV* lesion volume, *PSI* phase synchrony index, *PLI* phase lag index.

Stroke LV was significantly negatively correlated with the ARSsp score (*ρ* =  − 0.69, *P* < 0.001, non-multiple comparison; Fig. 7a). Thus, LV could be a confounding factor in correlation analyses of the PSI/PLI with the ARSsp scores. Nonparametric partial correlation analysis revealed that the F7T5-PSIs (β1/β2/γ) were not significantly correlated with ARSsp scores (β1: *ρ* =  − 0.32, *P* = 0.082; β2: *ρ* =  − 0.34, *P* = 0.063; γ: *ρ* =  − 0.30, *P* = 0.103, before correction). By contrast, the F7F8-PSI (β1: *ρ* = 0.55, *P* = 0.014), the F8T6-PSI (δ: *ρ* =  − 0.52, *P* = 0.021) and the F8T6-PLI (β1: *ρ* = 0.63, *P* = 0.003) were significantly correlated with ARSsp scores after correction for the LV effect (*P* < 0.05, FDR corrected). Figure 7b summarizes the outcome of the analyses in this study.

In summary, the interhemispheric frontofrontal PSI was positively correlated with aphasia scores, whereas the right frontotemporal PSI was negatively correlated with aphasia scores, showing a significant difference of PSI values in the same frequency bands compared with healthy controls. In contrast, the F8T6-PLI was positively correlated with aphasia scores with no significant difference to healthy controls.

## Discussion

In patients with subacute aphasia, the decreased interhemispheric frontofrontal PSI (β band) positively correlated with the aphasia score, whereas the increased right frontotemporal PSI (δ band) negatively correlated with the aphasia score independent of stroke LV. Moreover, these PSIs showed significant differences with healthy controls in the same frequency bands. These results suggest that aphasia in subacute stroke is associated with alterations in the two distinct phase synchrony networks.

In the synchrony analysis of EEG data, we have to consider the possibility of the spurious synchrony due to artifacts^21,23^. Therefore, we performed several complementary analyses. We assessed PSIs of spatially adjacent electrode pairs (F3F4/F3P3/F4P4-PSIs) that are not located in the motor language-related regions. If the effects of artifacts such as common reference problem and volume conduction effect caused spurious synchrony rather than true synchrony, the F3F4/F3P3/F4P4-PSIs would give the same results as the F7F8/F7T5/F8T6-PSI. However, the results of the F3F4/F3P3/F4P4-PSIs suggested this is not the case. The synchrony in the F7T5-PSI observed in wide frequency range (β1 to γ; Fig. 2e) was considered to be spurious because the partial correlation analysis with LV as another variable revealed no significant correlation with ARSsp scores. Furthermore, the global PSIs that also could be influenced by the above potential artifacts, showed no significant correlation with the ARSsp scores. Thus, these examinations of the results for artifact suggest that this association is attributed to true synchrony rather than spurious synchrony.

We also evaluated the PLI, a more conservative synchrony measure than the PSI^22^. In our previous study for large-scale synchrony, the PLI showed a similar correlation pattern to the PSI^24,25^. In this study, however, the F7F8-PLI and the F7T5-PLI showed no significant correlation with ARSsp scores. The F8T6-PLI was correlated with the ARSsp scores with no significant difference to healthy controls. The PLI may be overly conservative to assess local synchrony between anatomically connected regions, underestimating the true synchrony with a near-zero phase lag.

In this study, the F7F8-PSI (β1 band) in patients with aphasia was significantly lower than that in healthy controls and significantly positively correlated with the ARSsp score. These findings were similar to the results of our previous report that focused on patients with hemiparesis^10^. The PSI (α band) covering the primary motor cortices in these patients was lower than that in healthy controls and positively correlated with the upper extremity motor function score. These results indicate that the decrease in homotopic interhemispheric PSIs may represent a common brain response in subacute stroke. Furthermore, in the current study, we found a significant correlation selectively in the β band. EEG β synchrony is associated with higher cognitive function^26^ and aphasia recovery^9^. These results suggest that network information reflecting motor or cognitive impairment may be mediated by distinct frequency bands.

The same trend of interhemispheric connectivity was observed in a previous fMRI study^6^. In that cross-sectional study with 132 subacute stroke patients, decreased interhemispheric homotopic FC was the most prominent change after stroke, and changes in interhemispheric FC showed strong associations with behavioral impairments, including aphasia. Furthermore, longitudinal normalization of decreased interhemispheric FC was associated with clinical recovery in another study^8^.

In our study, adjacent (but not motor language-related) PSI (F3F4-PSI) and EEG power (F7F8-wP) were not useful to evaluate post-stroke neural network alteration. The F7F8-PLI showed no significant correlation with ARSsp scores probably because the PLI was overly conservative to assess local synchrony between anatomically connected regions, thereby underestimating the true synchrony with a near-zero phase lag. In addition, a partial correlation analysis revealed that the correlation between the F7F8-PSI and ARSsp scores was independent of the stroke LV. Altogether, the decreased F7F8-PSI reflects decreased FC between the bilateral inferior frontal lobes (motor language area and its right-sided homotopic region) and may represent interhemispheric brain network alternation in subacute stroke. Thus, the F7F8-PSI (β1 band) may be a biomarker in post-stroke aphasia.

In previous studies, left frontotemporal FC was associated with the aphasia score^17,18^. Although the F7T5-PSI was negatively correlated with the ARSsp score in the β1, β2, and γ bands, the F7T5-PSI (β1 and β2) values of patients with aphasia did not differ significantly from those of healthy controls. Conversely, the F7T5-PSI significantly correlated with the LV in the β1, β2, and γ bands. Because LV negatively correlated with the ARSsp score, the correlation of the F7F8-PSI with the ARSsp score was likely induced by a confounding LV effect, rather than by true EEG synchrony. In fact, partial correlation analysis revealed that the F7T5-PSI showed no significant correlation with the ARSsp score after correction for the LV effect. This result coincided with our previous studies, in that, when all electrodes were located within the ipsilesional hemisphere, the PSI value was inflated by spurious synchrony due to an artifact (volume-conduction effect)^10,24^. Thus, the F7T5-PSI is not appropriate for assessing the intrahemispheric brain network within the ipsilesional hemisphere. The adjacent PSI (F3P3-PSI), EEG power (F7T5-wP), global PSI (left Intrah-PSI), and the F7T5-PLI were not useful to evaluate post-stroke neural network alteration in the left hemisphere.

The F8T6-PSI (δ band) in patients with aphasia was significantly higher than that in healthy controls and significantly negatively correlated with the ARSsp score. Regarding contralesional intrahemispheric EEG synchrony, our previous study demonstrated that the contralesional PSI (θ band) centered on the primary motor cortex of patients with hemiparesis was higher than that of healthy controls^10^. Similar findings were reported in a longitudinal fMRI study showing that FC of the right homotopic language area was activated in the subacute stage and normalized in the chronic stage^27^.

In a cross-sectional fMRI study, a decrease in interhemispheric FC was accompanied by an increase in intrahemispheric FC^6^. In our EEG results, a similar segregation of interhemispheric/intrahemispheric synchrony was observed. In contrast to the F7F8-PSI, the F8T6-PSI negatively correlated with the ARSsp score. In another EEG study, increased δ band activity was associated with reduced interhemispheric connectivity^28^. An fMRI study reported a trend of inverse association between the language network FC and verbal executive function (higher FC exhibits a larger deficit)^29^. These findings suggest that the negative correlation between the increased F8T6-PSI and the ARSsp score represents right intrahemispheric inhibition due to interhemispheric disinhibition by the left hemisphere. Another explanation may be that increased δ band synchrony reflects compensation for a greater deficit without being sufficient to improve the clinical status. With respect to the band frequency, δ band synchrony mediates large-scale cortical integration^30^. In contrast, as an EEG study recently reported a negative correlation between δ band coherence of the bilateral primary motor cortices and the motor score in patients with stroke^31^, δ band synchrony may also relate to functional recovery from hemiparesis.

The adjacent PSI (F4P4-PSI), EEG power (F8T6-wP) and global PSI (right Intrah-PSI) were not useful. In addition, the result of the F8T6-PLI was inconsistent because values of the F8T6-PLI showed no significant difference to healthy controls. The correlation between the F8T6-PSI and the ARSsp score remained significant after removing the effect of stroke LV. Taken together, an increased F8T6-PSI is considered to reflect real increase in FC between the right frontotemporal lobes, and although it remains to be elucidated whether this increase is a compensatory or simple reaction to the disinhibition, the F8T6-PSI (δ band) may be a biomarker that can be used to better understand the pathophysiologic mechanism in the contralesional hemisphere.

Notably, the method suggested in this study is clinically feasible because the less than 5-min resting-state EEG recording based on the standard International 10–20 system can be easily performed in most medical facilities. In addition, our findings from a moderate number of patients (n = 31) with common etiology (ischemic stroke) and stroke lesion (cortical lesion including frontal lobe) might be generalizable to a similar stroke population.

This study has some limitations. First, it was difficult to completely eliminate the effects of artifacts such as common reference problem and volume conduction effect. However, examination of the spurious synchrony indicates the usefulness of our analysis. Second, the low electrode density was disadvantageous owing to limited spatial resolution. Third, we had a limited range of choice in electrode pairs. That was a reason why we focused on the inferior frontal lobes (F7 and F8) and speech subscores in SLTA. Fourth, all patients were native Japanese speakers. Language factors intrinsic to Japanese may hinder the expansion of our findings to other populations. Finally, the causality of the correlations between PSIs and ARSsp scores could not be determined from this study.

## Conclusions

This study provides empirical evidence that post-stroke aphasia has an aspect of a network disorder. Our results suggest two different PSIs linked to the right inferior frontal lobe, namely the frontofrontal PSI and left frontotemporal PSI, reflect the segregation of interhemispheric/intrahemispheric networks. Furthermore, the existence of distinct frequency bands that may mediate specific network information is a unique feature of EEG synchrony analysis. Although further research is needed to confirm whether PSIs are useful in patients with receptive aphasia, our method is adaptable for clinical use. In conclusion, our novel findings suggest that the PSI could be a useful tool to better understand pathophysiological mechanisms associated with subacute stroke and serve as a potential biomarker of cognitive and motor functions.

## Methods

### Ethical approval

The experimental protocols of this study were approved by the Institutional Review Boards of RIKEN and Morinomiya Hospital and adhered to the tenets of the Declaration of Helsinki. All participants or their proxies provided written informed consent. In case of difficulties in understanding explanations and/or in signing their name due to aphasia and/or paralysis, the patient’s proxy provided written informed consent.

### Participants

All patients enrolled in this study were recruited from a cohort of inpatients admitted to the Kaifukuki (convalescent) rehabilitation ward^32^ at Morinomiya Hospital. The inclusion criteria were as follows: (1) age ≥ 20 years, (2) first episode of unilateral cortical ischemic stroke with a left frontal lobe lesion, irrespective of temporal/parietal lobe involvement on MRI, (3) presence of aphasia at the time of admission, (4) right-handedness, (5) Japanese speaker, and (6) interval since the onset of stroke > 2 weeks. The exclusion criteria were as follows: (1) a medical history of psychiatric or neurological disorders, such as epilepsy, dementia, and Parkinson’s disease, (2) lack of a left frontal lesion, and (3) skull defects. Age- and sex-matched healthy participants who had no history of psychiatric or neurological disorders were also recruited as a healthy control group. Thirty-one patients and 24 control participants were enrolled in the study.

### Clinical assessments

We used the SLTA to assess patients with aphasia^20^. The SLTA, commonly used in Japan^33–35^, was designed for profiling the level of aphasia of patients and consists of 26 subscores (Supplementary Table S41 online). For quantitative analysis, Hasegawa et al. developed the Aphasia Rating Scale (ARS) based on factorial analysis of the SLTA data obtained from 313 patients with aphasia^19^. They categorized 19 subscores of the SLTA into three functions: writing (seven items), speech (seven items), and comprehension (five items). In this study, we focused on the expressive function of patients with left frontal lobe lesions, more specifically on speech function, because some patients with frontal lobe lesions had difficulty in writing owing to hemiparesis. According to the 19-channel electrode setting, we focused on the inferior frontal lobes (electrodes F7 and F8; Fig. 1a) and speech score because the posterior language system is too widely distributed to be addressed appropriately with this setting^36^. The seven items of the SLTA, comprising the ARSsp, are object naming, description of pictures, description of four-panel cartoons, kanji word reading, single kana letter reading, kana word reading, and short sentence reading. We allocated 10 points to each item and summed these to assess the severity of patients’ expressive function deficit (range: 0–70, with *a lower score indicating a more severe deficit*). Assessment of the SLTA of patients was performed by trained speech therapists. We also assessed patients’ general neurological deficit using the National Institutes of Health Stroke Scale (range: 0–42). We assessed cognitive function of healthy control participants using the Mini Mental State Examination. All clinical assessments were performed by trained staff members who were blinded to the patients’ PSI values.

### EEG recording, data processing, and PSI computation

We recorded scalp EEGs using a NeuroFax EEG 1224 system (Nihon Kohden Co., Tokyo, Japan) with an online bandpass filtering between 0.53 and 120 Hz and a sampling rate of 500 Hz. EEG data were recorded from 19 Ag/AgCl electrodes located according to the International 10–20 system, with a ground electrode located at the center of the forehead. Participants were asked to rest in the supine position and alternately open and close their eyes for 30 s under each condition, comprising five sessions. We used the eyes-closed condition data (30 s × 5 sessions) because the eyes-opened condition data were subject to motion artifacts of the eyelids and eyeballs.

EEG voltages were re-referenced to the average of bilateral earlobe signals. After offline bandpass filtering (0.3–50 Hz), data points at which the voltage exceeded ± 200 μV were rejected. Then, the complex Morlet wavelet transform was applied to the remaining data points, to extract the instantaneous phase and amplitude. The cutoff interval length of the Gaussian window, “n_co_,” was four cycles^11^. The PSI at time-point *tau* was defined by the following Eq. (1):

1 $$PSI\left( \tau \right) = \left| {\frac{1}{T}\mathop \sum \limits_{{t = \tau - \frac{T}{2}}}^{{t = \tau + \frac{T}{2}}} {\text{exp}}\left\{ {i\left( {\theta_{t}^{m} - \theta_{t}^{n} } \right)} \right\}} \right|,$$

where *T* is the number of time-points, which corresponds to eight cycles of the center frequency of the complex Morlet wavelet, *i* denotes the imaginary unit, $\uptheta _{t}^{m}$ and $\theta_{t}^{n}$ indicate the instantaneous phases of the *m*th and *n*th electrodes at the time-point *t*. PSIs, which range from zero (no synchrony) to one (perfect synchrony), were obtained for every time-point (2-ms steps) and frequency (1-Hz steps) and averaged for each participant within six frequency bands, as follows: δ (1–3 Hz), θ (4–7 Hz), α (8–13 Hz), β1 (14–19 Hz), β2 (20–30 Hz), and γ (31–45 Hz). Details of EEG recording and data processing are fully described in our previous report^10^.

To assess the interhemispheric frontofrontal network, the F7F8-PSI was computed as the PSI between the F7 and F8 electrodes (Fig. 1a) that were placed over the left inferior frontal lobe (F7: motor language area) and right homotopic region (F8). To assess the left intrahemispheric frontotemporal network, the PSI between the inferior frontal lobe (F7) and posterior temporal lobe (T5) was computed (F7T5-PSI). Similarly, the right frontotemporal PSI (F8T6-PSI) was computed. To assess the effect of artifacts, we also evaluated the spatially adjacent electrode pairs (F3F4/F3P3/F4P4) that were not located in the motor language-related regions (Fig. 1b). Although the F7F8/T7T5/F8T6-PSI and the F3F4/F3P3/F4P4-PSIs should be influenced by artifacts in the same way, the latter would not be associated with network changes in patients with aphasia. In addition, we evaluated global synchrony by the Intrah-PSI. Left/right Intrah-PSIs were computed as the average of the local PSIs across all intrahemispheric electrode pairs of left/right hemispheres, respectively.

We evaluated the PLI for comparison with the PSIs as a tool to assess brain networks. The PLI (τ) was defined by the following Eq. (2):

2 $$PLI\left( \tau \right) = \left| {\frac{1}{T}\mathop \sum \limits_{{t = \tau - \frac{T}{2}}}^{{t = \tau + \frac{T}{2}}} {\text{sign}}\left( {\theta_{t}^{m} - \theta_{t}^{n} } \right)} \right|,$$

where *T* is the number of time points (8 cycles of the central frequency), the sign indicates a signum function, which is defined as:

$${\text{sign}}\left( {\text{x}} \right){: = }\left\{ {\begin{array}{*{20}c} { - 1{\text{ if x < 0,}}} \\ {\text{ 0 if x = 0,}} \\ {{\text{ 1 if x > 0}}{.}} \\ \end{array} } \right\}$$

We also evaluated the EEG wP for comparison with the PSIs. The average wP of the *m*th electrode was computed as follows using Eq. (3):

3 $$wP\left( m \right) = \frac{1}{{T_{tot} }}\mathop \sum \limits_{\tau } \left( {A_{\tau }^{m} } \right)^{2} ,$$

where $A_{\tau }^{m}$ means instantaneous amplitudes of the *m*th electrode at the time-point *tau* and *T*_*tot*_ represents the total number of time-points. wP was also averaged for each participant within the six frequency bands. We calculated the difference in the EEG power of the same electrode locations for comparison with the PSI representing the difference of the EEG phases between the two electrodes. For example, the F7F8-wP was calculated by subtracting wP(F8) from wP(F7). F7T5-wP and F8T6-wP were calculated in the same way.

The PSI, PLI, and wP were computed using programs developed in MATLAB (MathWorks Inc., Natick, MA).

### MRI acquisition and stroke LV computation

MRI was conducted using a 1.5-T scanner (Achieva, Philips Medical Systems, Best, The Netherlands) for all patients. T2-weighted images (turbo spin-echo, reconstructed voxel size = 0.45 × 0.45 × 5.00 mm^3^) and three-dimensional T1-weighted images (turbo field-echo, reconstructed voxel size = 0.94 × 0.94 × 1.00 mm^3^) were obtained to cover the whole brain. To compute stroke LV, lesions of each patient were manually drawn on the T2-weighted images, and they were spatially normalized to the Montreal Neurological Institute stereotaxic space, using the Clinical Toolbox^37^ in SPM8^38^ (Wellcome Trust Centre for Neuroimaging, London, UK). Details of the MRI acquisition conditions are fully described in our previous report^10^.

### Statistical analysis

The Shapiro‒Wilk test was used to assess the normality of variables. A nonparametric statistical test was conducted for variables with skewed distribution. The Mann‒Whitney U test was used to compare the medians of unpaired nonparametric variables between patients and healthy controls. Spearman’s rank correlation analysis was used for nonparametric correlation analysis. To control for multiple comparisons, FDR correction (Benjamini and Hochberg method) was applied^39^, considering the number of tested hypotheses (the number of frequency bands × the number of electrode pairs). To evaluate the effects of LV as a confounding factor, nonparametric Spearman’s rank order partial correlation analysis^40^ was performed (control variable: LV). A two-sided *P* value < 0.05 was considered to indicate statistical significance. All statistical analyses were performed using IBM SPSS software (version 25.0, IBM Corp., Armonk, NY).

## Supplementary Information

Supplementary Tables.

## Data Availability

The authors declare that all data generated or analyzed during this study are included in this published article and its Supplementary information files.
