# Supplementary material for: Association between aphasia severity and brain network alterations after stroke assessed using the electroencephalographic phase synchrony index
Source: Sci Rep. 2021 Jun 14;11:12469. doi: 10.1038/s41598-021-91978-7 (PMC8203681; doi:10.1038/s41598-021-91978-7)
Supplement: Supplementary file 1 — Supplementary Tables. [file 41598_2021_91978_MOESM1_ESM.pdf]

Supplementary information

Association between aphasia severity and brain network alterations after stroke assessed using the electroencephalographic phase synchrony index

Teiji Kawano, Noriaki Hattori, Yutaka Uno, Megumi Hatakenaka, Hajime Yagura, Hiroaki Fujimoto, Michiko Nagasako, Hideki Mochizuki, Keiichi Kitajo, Ichiro Miyai

Supplementary tables

Table S1. Demographic and clinical characteristics of each patient
Table S2. Demographic and clinical characteristics of each healthy control participant
Table S3. F7F8-PSI value of each patient
Table S4. F7T5-PSI value of each patient
Table S5. F8T6-PSI value of each patient
Table S6. F7F8-PSI value of each healthy control participant
Table S7. F7T5-PSI value of each healthy control participant
Table S8. F8T6-PSI value of each healthy control participant
Table S9. F3F4-PSI value of each patient
Table S10. F3P3-PSI value of each patient
Table S11. F4P4-PSI value of each patient
Table S12. F3F4-PSI value of each healthy control participant
Table S13. F3P3-PSI value of each healthy control participant
Table S14. F4P4-PSI value of each healthy control participant
Table S15. left Intrah-PSI value of each patient
Table S16. right Intrah-PSI value of each patient
Table S17. left Intrah-PSI value of each healthy control participant
Table S18. right Intrah-PSI value of each healthy control participant

Table S19. F7F8-PLI value of each patient
Table S20. F7T5-PLI value of each patient
Table S21. F8T6-PLI value of each patient
Table S22. F7F8-PLI value of each healthy control participant
Table S23. F7T5-PLI value of each healthy control participant
Table S24. F8T6-PLI value of each healthy control participant
Table S25. F7F8-wP value of each patient
Table S26. F7T5-wP value of each patient
Table S27. F8T6-wP value of each patient
Table S28. F7F8-wP value of each healthy control participant
Table S29. F7T5-wP value of each healthy control participant
Table S30. F8T6-wP value of each healthy control participant
Table S31. Comparison of the PSI values between patients and healthy control participants
Table S32. Results of correlation analyses between the PSI and the ARSsp score
Table S33. Comparison of the Intrah-PSI values between patients and healthy control participants
Table S34. Results of correlation analyses between the Intrah-PSI and the ARSsp score
Table S35. Comparison of the PLI values between patients and healthy control participants
Table S36. Results of correlation analyses between the PLI and the ARSsp score
Table S37. Comparison of the wP values between patients and healthy control participants
Table S38. Results of correlation analyses between the wP and the ARSsp score
Table S39. Results of correlation analyses between the PSI and the LV
Table S40. Results of correlation analyses between the PLI and the LV
Table S41. Contents of the SLTA

Abbreviations: ARSsp, Aphasia Rating Scale speech; Intrah, intrahemispheric; LV, lesion volume; PLI, phase lag index; PSI, phase synchrony index; SLTA, Standard Language Test of Aphasia; wP, wavelet power.

Supplementary table S1. Demographic and clinical characteristics of each patient

ID	Age	Sex	EEG recording after stroke onset (days)	NIHSS score (42)	ARSsp score (70)	LV (mm^3)
1	78	W	32	10	2	69,424
2	53	M	63	19	2	205,520
3	90	M	42	10	12.5	108,712
4	36	M	25	5	59	74,568
5	68	M	24	4	68	58,416
6	80	W	59	3	54.5	25,816
7	63	M	28	9	26.5	93,176
8	85	W	43	2	61.5	23,528
9	75	M	43	6	64	36,464
10	69	M	40	12	0	187,472
11	58	M	62	17	3.5	139,920
12	63	M	32	3	52	59,704
13	81	M	41	3	12	33,480
14	40	M	19	1	67	68,800
15	69	M	37	18	0	293,376
16	61	M	44	3	37	103,216
17	83	W	26	8	2	49,944
18	69	M	30	2	55.5	41,304
19	77	M	43	6	8	117,904
20	55	M	27	1	68	14,584
21	48	M	31	0	70	14,504

22	65	M	22	1	62.5	51,568
23	80	M	30	2	68	11,968
24	81	M	43	17	0	66,888
25	75	W	38	9	47	97,208
26	71	W	31	11	41.5	33,464
27	73	W	33	2	47	10,920
28	68	M	28	6	0	161,976
29	55	M	37	1	62	21,768
30	58	M	27	4	51	56,040
31	81	M	70	7	10.5	87,680

Numbers in parentheses refer to the maximum score possible on each clinical scale.

Abbreviations: ARSsp, Aphasia Rating Scale speech; EEG, electroencephalography; ID, patient identification number; LV, lesion volume; M, Man; NIHSS, National Institutes of Health Stroke Scale; W, Woman.

Supplementary table S2. Demographic and clinical characteristics of each healthy control participant

ID	Age	Sex	Handedness	MMSE score (30)
1	57	W	right	27
2	72	W	right	30
3	72	M	right	26
4	65	W	right	30
5	73	M	right	30
6	61	W	right	30
7	73	W	right	30
8	75	M	right	30
9	71	W	right	30
10	55	W	right	27
11	66	W	right	30
12	73	M	right	30
13	67	M	right	27
14	65	M	right	28
15	58	M	right	30
16	62	M	right	30
17	58	M	right	30
18	67	M	right	29
19	59	M	right	26
20	71	M	right	30
21	80	M	right	30

22	61	W	right	30
23	56	W	right	30
24	67	M	right	29

Numbers in parentheses refer to the maximum score possible on each clinical scale.

Abbreviations: ID, patient identification number; M, Man; MMSE, Mini Mental State Examination; W, Woman.

Supplementary table S3. F7F8-PSI value of each patient

ID	δ	θ	α	β1	β2	γ
1	0.447687	0.419606	0.492956	0.359948	0.380767	0.384638
2	0.436608	0.484325	0.618433	0.365759	0.363998	0.383609
3	0.479322	0.456986	0.753074	0.478801	0.397132	0.381745
4	0.388104	0.501690	0.673076	0.502216	0.449812	0.447847
5	0.402014	0.384571	0.597037	0.451916	0.400577	0.370668
6	0.376120	0.400495	0.406253	0.367611	0.352278	0.366648
7	0.386386	0.502432	0.518004	0.388900	0.394698	0.435917
8	0.377344	0.477405	0.537715	0.401645	0.383558	0.380751
9	0.400460	0.590499	0.628719	0.429609	0.381401	0.396478
10	0.719220	0.596060	0.620450	0.391311	0.354130	0.370423
11	0.362261	0.456491	0.514315	0.351697	0.369239	0.357567
12	0.378785	0.378830	0.570975	0.460914	0.413436	0.418895
13	0.411507	0.428017	0.517978	0.368967	0.358035	0.407376
14	0.379088	0.372046	0.519456	0.395761	0.368623	0.370900
15	0.408845	0.401108	0.403841	0.339986	0.352101	0.360372
16	0.387364	0.428678	0.485804	0.359749	0.374789	0.427207
17	0.372130	0.362389	0.447416	0.375308	0.404811	0.455221
18	0.521513	0.386905	0.462727	0.383235	0.410802	0.430865
19	0.550839	0.417645	0.494461	0.360278	0.354755	0.343302
20	0.397889	0.410911	0.656133	0.476643	0.362745	0.397650
21	0.382730	0.496310	0.765117	0.482720	0.439136	0.438047

22	0.451929	0.395128	0.446399	0.406111	0.341682	0.366727
23	0.401480	0.390040	0.570712	0.365986	0.363979	0.356873
24	0.368944	0.372699	0.396744	0.363480	0.369148	0.353626
25	0.392406	0.366665	0.442052	0.396216	0.394222	0.415979
26	0.396826	0.380466	0.375975	0.365530	0.365509	0.373282
27	0.410631	0.374832	0.440755	0.359494	0.359389	0.363993
28	0.384072	0.419158	0.494183	0.403006	0.407320	0.432277
29	0.392174	0.374707	0.483541	0.423563	0.429034	0.393514
30	0.347594	0.359375	0.403573	0.341011	0.362862	0.372945
31	0.376663	0.567907	0.659725	0.349387	0.377290	0.371683

Abbreviations: ID, patient identification number; PSI, phase synchrony index.

Supplementary table S4. F7T5-PSI value of each patient

ID	δ	θ	α	β1	β2	γ
1	0.563229	0.457555	0.440717	0.446074	0.495182	0.456528
2	0.417909	0.595573	0.666002	0.544585	0.492532	0.467828
3	0.438178	0.410912	0.568501	0.449479	0.479569	0.440564
4	0.434720	0.449946	0.507439	0.449702	0.450303	0.443070
5	0.390039	0.355059	0.548350	0.397540	0.399790	0.400054
6	0.407573	0.386221	0.381771	0.364851	0.394601	0.464013
7	0.372129	0.397393	0.398420	0.355667	0.381974	0.394461
8	0.347759	0.356403	0.438363	0.345838	0.362472	0.395009
9	0.427662	0.397370	0.397796	0.388541	0.443305	0.463576
10	0.687367	0.518778	0.564026	0.570000	0.592750	0.602347
11	0.388683	0.437388	0.440246	0.413061	0.465149	0.474511
12	0.377041	0.363390	0.464748	0.399014	0.360077	0.381711
13	0.431424	0.402454	0.504250	0.406527	0.407273	0.437749
14	0.354226	0.421951	0.429953	0.408079	0.429510	0.443747
15	0.449426	0.427704	0.519464	0.487149	0.495103	0.459880
16	0.413814	0.459147	0.485565	0.448085	0.474208	0.486148
17	0.339752	0.351471	0.473802	0.457185	0.475639	0.498113
18	0.481277	0.444872	0.451494	0.382777	0.415872	0.440025
19	0.368367	0.354641	0.477713	0.416459	0.407716	0.398502
20	0.436816	0.419545	0.490520	0.405095	0.387300	0.417436
21	0.389170	0.409152	0.501180	0.366501	0.398394	0.406786

22	0.427032	0.389172	0.433977	0.408026	0.347006	0.367717
23	0.481969	0.407195	0.514461	0.393687	0.420313	0.383299
24	0.403333	0.365728	0.410685	0.439508	0.460825	0.465231
25	0.455103	0.407300	0.460404	0.385948	0.441623	0.517307
26	0.423559	0.422320	0.406978	0.367106	0.377463	0.391192
27	0.475803	0.415154	0.483097	0.412801	0.394271	0.396856
28	0.427334	0.390195	0.425876	0.436289	0.499129	0.546188
29	0.457965	0.479262	0.519222	0.444154	0.468080	0.436224
30	0.306627	0.352643	0.406773	0.390573	0.420455	0.409102
31	0.425599	0.524126	0.562840	0.399855	0.460630	0.459677

Abbreviations: ID, patient identification number; PSI, phase synchrony index.

Supplementary table S5. F8T6-PSI value of each patient

ID	δ	θ	α	β1	β2	γ
1	0.631035	0.623207	0.727617	0.516988	0.465538	0.427756
2	0.591464	0.582293	0.572572	0.451945	0.416513	0.415881
3	0.557967	0.533642	0.685844	0.462285	0.421259	0.441130
4	0.538887	0.592988	0.661257	0.541231	0.572394	0.612865
5	0.446389	0.438786	0.617689	0.444292	0.428023	0.411410
6	0.380632	0.388139	0.364405	0.369076	0.346532	0.364385
7	0.576157	0.488911	0.452658	0.396366	0.472982	0.597204
8	0.510006	0.490315	0.542059	0.414927	0.367940	0.393592
9	0.472835	0.431641	0.423691	0.376009	0.413716	0.450104
10	0.617111	0.545163	0.531625	0.405978	0.463653	0.535220
11	0.524621	0.421835	0.572724	0.384399	0.373720	0.358815
12	0.530934	0.497465	0.472759	0.416102	0.406877	0.420953
13	0.500398	0.461385	0.542465	0.380682	0.417698	0.449844
14	0.435790	0.481176	0.532633	0.456353	0.403224	0.406570
15	0.654901	0.682663	0.676052	0.499962	0.435750	0.397119
16	0.537751	0.518876	0.482667	0.438082	0.499084	0.559246
17	0.677801	0.567298	0.490394	0.415970	0.436950	0.491705
18	0.478895	0.428152	0.449859	0.389155	0.502467	0.662316
19	0.673755	0.537029	0.607571	0.395484	0.378879	0.384226
20	0.502183	0.564758	0.586400	0.519177	0.512651	0.544361
21	0.399208	0.420079	0.663654	0.375505	0.381452	0.405800

22	0.590470	0.584004	0.560366	0.487131	0.408649	0.423218
23	0.533121	0.541514	0.695265	0.514231	0.445693	0.369585
24	0.562189	0.503610	0.515463	0.401618	0.369911	0.353986
25	0.416213	0.420445	0.453476	0.377479	0.402675	0.464523
26	0.603590	0.472704	0.433861	0.389987	0.377524	0.392508
27	0.652153	0.614670	0.544480	0.377463	0.378342	0.399493
28	0.489223	0.527827	0.536021	0.420814	0.457339	0.562712
29	0.409723	0.451763	0.566497	0.524573	0.499856	0.460590
30	0.502632	0.529617	0.524988	0.395913	0.399002	0.465955
31	0.491925	0.491128	0.471675	0.363500	0.418170	0.425259

Abbreviations: ID, patient identification number; PSI, phase synchrony index.

Supplementary table S6. F7F8-PSI value of each healthy control participant

ID	δ	θ	α	β1	β2	γ
HC1	0.365545	0.478731	0.790224	0.477247	0.400728	0.394200
HC2	0.414811	0.386438	0.474853	0.397550	0.373481	0.381301
HC3	0.392924	0.358683	0.495503	0.416280	0.381258	0.381407
HC4	0.370121	0.398608	0.429433	0.404126	0.414933	0.385104
HC5	0.405886	0.421480	0.459472	0.419935	0.428943	0.424938
HC6	0.374713	0.356614	0.403058	0.364153	0.374817	0.386804
HC7	0.517495	0.427932	0.518901	0.459306	0.444043	0.411043
HC8	0.375045	0.493579	0.688592	0.489265	0.424884	0.370532
HC9	0.441638	0.370030	0.475970	0.393729	0.387096	0.338441
HC10	0.408340	0.485296	0.773351	0.583348	0.439443	0.456642
HC11	0.367402	0.349207	0.458982	0.412969	0.393935	0.384922
HC12	0.361347	0.412713	0.626418	0.371901	0.363653	0.355869
HC13	0.540474	0.420009	0.406565	0.422576	0.464999	0.487872
HC14	0.382582	0.506974	0.740346	0.510597	0.425916	0.382985
HC15	0.397133	0.413283	0.699880	0.415449	0.373863	0.386624
HC16	0.322421	0.427921	0.521800	0.382814	0.392459	0.430411
HC17	0.531318	0.414994	0.500652	0.430876	0.398827	0.361776
HC18	0.372434	0.387609	0.539595	0.371812	0.395676	0.374244
HC19	0.375673	0.376970	0.420614	0.367685	0.399171	0.397430
HC20	0.350385	0.385279	0.575388	0.423653	0.407100	0.407010
HC21	0.391875	0.363944	0.579080	0.456659	0.439485	0.381419

HC22	0.365330	0.439106	0.667965	0.445729	0.423313	0.383512
HC23	0.369819	0.422098	0.690713	0.403556	0.388465	0.381305
HC24	0.377397	0.478914	0.682814	0.416518	0.385456	0.347827

Abbreviations: HC, healthy control; ID, patient identification number; PSI, phase synchrony index.

Supplementary table S7. F7T5-PSI value of each healthy control participant

ID	δ	θ	α	β1	β2	γ
HC1	0.460786	0.440142	0.520864	0.429671	0.477135	0.465155
HC2	0.554507	0.523353	0.545372	0.466163	0.433518	0.442393
HC3	0.365837	0.373799	0.443333	0.350755	0.364753	0.385848
HC4	0.432285	0.495292	0.472209	0.423507	0.400123	0.396621
HC5	0.410053	0.415925	0.416606	0.397519	0.393012	0.436172
HC6	0.353800	0.410049	0.443334	0.416396	0.395385	0.407816
HC7	0.504297	0.447534	0.443227	0.408097	0.416902	0.427420
HC8	0.373988	0.426522	0.626205	0.405631	0.376482	0.380223
HC9	0.436958	0.420190	0.422036	0.410524	0.414780	0.374764
HC10	0.505727	0.455582	0.578571	0.447640	0.395586	0.414537
HC11	0.432171	0.402743	0.451181	0.380504	0.410798	0.371576
HC12	0.378613	0.377705	0.535984	0.361247	0.383914	0.380933
HC13	0.508714	0.463396	0.451040	0.461932	0.488291	0.541564
HC14	0.428542	0.460671	0.475687	0.410728	0.405293	0.368303
HC15	0.380655	0.357650	0.542842	0.372614	0.367236	0.368096
HC16	0.404460	0.455939	0.505816	0.442943	0.449336	0.487394
HC17	0.486789	0.462912	0.419912	0.375437	0.406380	0.360329
HC18	0.385073	0.367942	0.481708	0.356218	0.384185	0.392123
HC19	0.471443	0.435932	0.427914	0.433337	0.436927	0.454529
HC20	0.365979	0.457397	0.532130	0.451802	0.454093	0.476907
HC21	0.386007	0.397997	0.426384	0.363550	0.394215	0.390690

HC22	0.485648	0.414476	0.486008	0.399813	0.407249	0.410559
HC23	0.452594	0.445764	0.471959	0.424294	0.400929	0.388343
HC24	0.377917	0.406731	0.611400	0.375009	0.390010	0.380049

Abbreviations: HC, healthy control; ID, patient identification number; PSI, phase synchrony index.

Supplementary table S8. F8T6-PSI value of each healthy control participant

ID	δ	θ	α	β1	β2	γ
HC1	0.486996	0.547135	0.595960	0.438014	0.432794	0.435136
HC2	0.495146	0.475094	0.524224	0.460672	0.450864	0.426764
HC3	0.559537	0.501331	0.551518	0.445456	0.436600	0.434212
HC4	0.479204	0.475335	0.446158	0.411777	0.413755	0.431350
HC5	0.449662	0.463881	0.442126	0.419597	0.405142	0.436839
HC6	0.399172	0.392603	0.452780	0.390795	0.379064	0.377662
HC7	0.500001	0.419471	0.405915	0.368251	0.388256	0.372596
HC8	0.433713	0.446350	0.633884	0.407405	0.377456	0.382413
HC9	0.370363	0.366306	0.418124	0.392991	0.381971	0.360425
HC10	0.453819	0.417756	0.569552	0.427648	0.389577	0.417492
HC11	0.443433	0.421449	0.416069	0.393783	0.416806	0.404371
HC12	0.364965	0.392766	0.601791	0.383935	0.378653	0.398074
HC13	0.527118	0.512916	0.486755	0.512351	0.544109	0.570155
HC14	0.465012	0.468567	0.492802	0.439155	0.408277	0.418002
HC15	0.428540	0.410326	0.530308	0.386716	0.386836	0.377040
HC16	0.385198	0.468149	0.515598	0.435298	0.462629	0.494319
HC17	0.600936	0.529875	0.543467	0.466906	0.459579	0.391402
HC18	0.405779	0.394101	0.456548	0.368340	0.384418	0.387405
HC19	0.424680	0.444721	0.489450	0.432735	0.427516	0.435002
HC20	0.352236	0.448523	0.501846	0.419778	0.410105	0.392565
HC21	0.368172	0.400219	0.554237	0.411445	0.427017	0.416576

HC22	0.423604	0.377085	0.453763	0.372463	0.400156	0.386292
HC23	0.474596	0.424490	0.493720	0.391120	0.377685	0.358149
HC24	0.427731	0.464032	0.639798	0.398553	0.402537	0.366154

Abbreviations: HC, healthy control; ID, patient identification number; PSI, phase synchrony index.

Supplementary table S9. F3F4-PSI value of each patient

ID	δ	θ	α	β1	β2	γ
1	0.629008	0.626892	0.650921	0.551653	0.557531	0.538924
2	0.586357	0.648061	0.755704	0.517048	0.463514	0.439605
3	0.585154	0.586336	0.862932	0.658374	0.536024	0.460802
4	0.652735	0.772031	0.833856	0.696634	0.602092	0.573368
5	0.505152	0.618229	0.838734	0.715966	0.608555	0.510846
6	0.579623	0.567925	0.563011	0.528274	0.548163	0.601975
7	0.634421	0.668158	0.626912	0.511112	0.504163	0.541440
8	0.519543	0.606130	0.633160	0.458437	0.396945	0.381260
9	0.681957	0.793114	0.803005	0.593850	0.547022	0.590531
10	0.902453	0.837096	0.843841	0.654839	0.562023	0.597092
11	0.593102	0.641979	0.664930	0.449617	0.445095	0.466723
12	0.469973	0.551928	0.794851	0.662131	0.537393	0.475164
13	0.507750	0.605300	0.649215	0.494989	0.445100	0.415041
14	0.555332	0.643074	0.738225	0.560189	0.443229	0.471336
15	0.612414	0.731356	0.636196	0.478497	0.439564	0.463422
16	0.490966	0.643498	0.745932	0.615456	0.591956	0.593911
17	0.615415	0.600724	0.652631	0.524455	0.518330	0.577794
18	0.384226	0.441836	0.570771	0.433287	0.460006	0.489959
19	0.538300	0.543640	0.662970	0.438391	0.418527	0.447113
20	0.627096	0.723696	0.839522	0.675993	0.497987	0.469947
21	0.649385	0.808814	0.904142	0.734333	0.693790	0.681649

22	0.408764	0.560357	0.618009	0.556321	0.430292	0.427414
23	0.616355	0.635946	0.768535	0.585824	0.538912	0.470121
24	0.473468	0.525402	0.462935	0.414939	0.420179	0.391673
25	0.524985	0.566027	0.611176	0.552791	0.515478	0.534252
26	0.462666	0.550797	0.582738	0.468165	0.463928	0.492465
27	0.679971	0.691599	0.729201	0.545462	0.503680	0.496825
28	0.521098	0.678232	0.756924	0.655189	0.641834	0.658776
29	0.556459	0.656770	0.773748	0.721666	0.715630	0.669568
30	0.594840	0.642398	0.684962	0.575945	0.568723	0.625890
31	0.524179	0.758245	0.832625	0.576576	0.544570	0.543773

Abbreviations: ID, patient identification number; PSI, phase synchrony index.

Supplementary table S10. F3P3-PSI value of each patient

ID	δ	θ	α	β1	β2	γ
1	0.664094	0.640478	0.649885	0.624686	0.621791	0.573110
2	0.520167	0.690491	0.735065	0.578907	0.536520	0.492671
3	0.558631	0.594513	0.824702	0.735565	0.662983	0.580764
4	0.648998	0.722908	0.735486	0.666965	0.647599	0.636555
5	0.489504	0.454270	0.615104	0.509418	0.513756	0.491932
6	0.598651	0.604844	0.501634	0.525810	0.574246	0.694366
7	0.472024	0.504218	0.483626	0.431135	0.479040	0.559339
8	0.428780	0.418227	0.475619	0.386220	0.376545	0.362574
9	0.547257	0.529235	0.516152	0.461476	0.446003	0.472843
10	0.693369	0.558029	0.594352	0.557535	0.580519	0.635805
11	0.418616	0.449213	0.569117	0.559162	0.535463	0.564355
12	0.526747	0.556872	0.590716	0.479179	0.505359	0.502233
13	0.404339	0.422471	0.499074	0.410408	0.385121	0.397770
14	0.551244	0.609499	0.617730	0.536369	0.466773	0.490530
15	0.521289	0.616879	0.647606	0.557136	0.539006	0.561541
16	0.488212	0.485153	0.526320	0.475727	0.510906	0.556059
17	0.563825	0.568992	0.640754	0.535863	0.536104	0.601428
18	0.453366	0.475566	0.515267	0.432187	0.462727	0.493666
19	0.566274	0.525551	0.647015	0.460832	0.418240	0.415149
20	0.568526	0.695646	0.694099	0.607767	0.527666	0.547422
21	0.658502	0.703858	0.703179	0.553159	0.523873	0.546834

22	0.530650	0.547711	0.619145	0.578316	0.513725	0.550192
23	0.616931	0.539095	0.562695	0.490489	0.514268	0.512324
24	0.457465	0.479605	0.532048	0.490421	0.447615	0.429333
25	0.629070	0.554422	0.583702	0.541233	0.580350	0.663185
26	0.530542	0.558818	0.563244	0.470542	0.470181	0.567608
27	0.695335	0.655999	0.697459	0.570976	0.536216	0.562582
28	0.577670	0.585033	0.576640	0.610781	0.642868	0.698370
29	0.604621	0.601952	0.647164	0.599971	0.628480	0.646193
30	0.397227	0.464229	0.532428	0.474971	0.533457	0.633249
31	0.520977	0.617083	0.654787	0.531549	0.602767	0.642314

Abbreviations: ID, patient identification number; PSI, phase synchrony index.

Supplementary table S11. F4P4-PSI value of each patient

ID	δ	θ	α	β1	β2	γ
1	0.677222	0.693123	0.813884	0.637733	0.561184	0.524068
2	0.653187	0.702518	0.681666	0.497629	0.420471	0.425862
3	0.648476	0.670840	0.875735	0.604584	0.481632	0.487723
4	0.687755	0.776331	0.771913	0.656347	0.625070	0.649553
5	0.548274	0.528471	0.696015	0.532373	0.493918	0.474452
6	0.542057	0.527747	0.451495	0.459857	0.548157	0.701212
7	0.708116	0.667688	0.578600	0.486598	0.490891	0.565477
8	0.565903	0.520580	0.606474	0.442704	0.394542	0.420796
9	0.580533	0.605193	0.592442	0.556596	0.494266	0.519081
10	0.780652	0.731448	0.687103	0.574300	0.578240	0.663741
11	0.537556	0.436548	0.633919	0.415745	0.373993	0.392909
12	0.660027	0.650448	0.610991	0.536577	0.518640	0.523713
13	0.542439	0.458605	0.498892	0.414254	0.426347	0.462603
14	0.540980	0.596545	0.640511	0.550147	0.428918	0.419568
15	0.785787	0.768173	0.814667	0.652724	0.571032	0.598115
16	0.625014	0.610632	0.581983	0.491496	0.497488	0.556541
17	0.773112	0.686033	0.667242	0.540086	0.528098	0.594796
18	0.483390	0.480434	0.585720	0.455363	0.527599	0.674734
19	0.735583	0.578747	0.630842	0.405714	0.378555	0.391888
20	0.679521	0.723158	0.781775	0.734881	0.662591	0.694025
21	0.629306	0.629255	0.626429	0.502752	0.476623	0.512603

22	0.724280	0.719136	0.706957	0.616853	0.552328	0.574093
23	0.601350	0.605269	0.708138	0.527979	0.508444	0.593633
24	0.554300	0.566862	0.572838	0.434978	0.410531	0.393204
25	0.623906	0.640177	0.607231	0.532034	0.502769	0.546611
26	0.649107	0.531789	0.524902	0.444281	0.466326	0.596756
27	0.780992	0.729159	0.756301	0.599790	0.560226	0.595726
28	0.512934	0.564333	0.570979	0.472845	0.470464	0.556412
29	0.545226	0.602465	0.696532	0.660130	0.629445	0.639561
30	0.528705	0.597786	0.628318	0.450480	0.438918	0.549406
31	0.588452	0.607505	0.573534	0.541164	0.603756	0.686875

Abbreviations: ID, patient identification number; PSI, phase synchrony index.

Supplementary table S12. F3F4-PSI value of each healthy control participant

ID	δ	θ	α	β1	β2	γ
HC1	0.751434	0.835618	0.939852	0.800259	0.706532	0.649340
HC2	0.495095	0.584503	0.737356	0.651027	0.574403	0.541129
HC3	0.516460	0.561576	0.720642	0.639957	0.601554	0.575125
HC4	0.599340	0.640556	0.668324	0.598618	0.540663	0.466413
HC5	0.560169	0.617164	0.638437	0.586891	0.566894	0.559373
HC6	0.458476	0.559170	0.661414	0.567211	0.509790	0.538183
HC7	0.772106	0.690521	0.776135	0.697392	0.650644	0.612374
HC8	0.387652	0.596250	0.841662	0.637800	0.573391	0.517717
HC9	0.543310	0.641801	0.765559	0.600635	0.547180	0.476121
HC10	0.663324	0.765171	0.902820	0.791975	0.645535	0.674642
HC11	0.712796	0.704879	0.788640	0.723456	0.639200	0.580105
HC12	0.453583	0.549544	0.757874	0.486089	0.424612	0.406748
HC13	0.646921	0.545932	0.538200	0.550469	0.569583	0.627562
HC14	0.562719	0.741623	0.860268	0.710911	0.620227	0.552133
HC15	0.422307	0.520007	0.766353	0.504748	0.435196	0.407629
HC16	0.514509	0.630164	0.730649	0.617138	0.587280	0.583253
HC17	0.497957	0.524429	0.615865	0.488024	0.432890	0.380472
HC18	0.452034	0.616768	0.780255	0.618561	0.582476	0.519117
HC19	0.554259	0.603277	0.616616	0.501898	0.489374	0.458393
HC20	0.518242	0.599020	0.761434	0.633518	0.584801	0.534159
HC21	0.476177	0.547395	0.819490	0.679581	0.624016	0.545557

HC22	0.516039	0.632932	0.834718	0.658663	0.577021	0.522645
HC23	0.617346	0.674196	0.846683	0.656421	0.540744	0.504117
HC24	0.673730	0.755293	0.871572	0.678970	0.569157	0.495747

Abbreviations: HC, healthy control; ID, patient identification number; PSI, phase synchrony index.

Supplementary table S13. F3P3-PSI value of each healthy control participant

ID	δ	θ	α	β1	β2	γ
HC1	0.661421	0.740835	0.768425	0.597072	0.560400	0.605404
HC2	0.639037	0.687129	0.750146	0.650047	0.590346	0.596502
HC3	0.465949	0.482231	0.535471	0.499438	0.503214	0.538923
HC4	0.612008	0.696611	0.674638	0.579103	0.502455	0.499791
HC5	0.583415	0.614807	0.589052	0.496779	0.456920	0.511996
HC6	0.540562	0.660830	0.724542	0.637987	0.545296	0.543501
HC7	0.637020	0.602750	0.593081	0.532435	0.523061	0.522534
HC8	0.370408	0.481971	0.645954	0.460996	0.419400	0.460400
HC9	0.576439	0.598064	0.583902	0.528177	0.462468	0.421975
HC10	0.656083	0.648767	0.700967	0.565792	0.461782	0.501558
HC11	0.685990	0.675647	0.672943	0.667973	0.619152	0.604214
HC12	0.477496	0.497410	0.648597	0.426253	0.401365	0.403179
HC13	0.498165	0.475981	0.521079	0.529353	0.556224	0.634005
HC14	0.608665	0.690370	0.684662	0.589814	0.550174	0.508576
HC15	0.409287	0.496875	0.676988	0.457758	0.417827	0.401300
HC16	0.470635	0.588481	0.603847	0.522393	0.540924	0.563182
HC17	0.497856	0.548188	0.582840	0.508546	0.475224	0.412255
HC18	0.388520	0.502865	0.561150	0.432094	0.410343	0.402771
HC19	0.560482	0.551156	0.566922	0.505808	0.497283	0.507846
HC20	0.501054	0.576751	0.695625	0.515577	0.457092	0.447929
HC21	0.487246	0.512619	0.739455	0.582065	0.517586	0.486675

HC22	0.515457	0.542072	0.647764	0.501057	0.448774	0.452522
HC23	0.623361	0.638439	0.770728	0.623750	0.463149	0.467863
HC24	0.529595	0.556299	0.650650	0.432579	0.422877	0.422576

Abbreviations: HC, healthy control; ID, patient identification number; PSI, phase synchrony index.

Supplementary table S14. F4P4-PSI value of each healthy control participant

ID	δ	θ	α	β1	β2	γ
HC1	0.711296	0.820062	0.839699	0.690829	0.644871	0.671556
HC2	0.589166	0.636991	0.708038	0.627118	0.568059	0.562635
HC3	0.589885	0.608488	0.695594	0.602768	0.589178	0.598922
HC4	0.620748	0.677111	0.645274	0.547311	0.486770	0.482086
HC5	0.483930	0.587731	0.556167	0.487067	0.448224	0.473649
HC6	0.445999	0.563932	0.646911	0.552995	0.461977	0.450111
HC7	0.629856	0.492829	0.433847	0.394846	0.412904	0.410636
HC8	0.388392	0.465151	0.620439	0.433613	0.398488	0.419886
HC9	0.549711	0.553895	0.601922	0.515052	0.453698	0.426742
HC10	0.653046	0.632439	0.704532	0.548368	0.437574	0.475964
HC11	0.661901	0.657326	0.657872	0.633186	0.573584	0.569693
HC12	0.483867	0.457838	0.620177	0.422393	0.383782	0.383434
HC13	0.564747	0.558240	0.538063	0.575920	0.599138	0.671010
HC14	0.659594	0.712038	0.736353	0.645256	0.558043	0.572930
HC15	0.510892	0.518930	0.712839	0.500129	0.457588	0.427244
HC16	0.412747	0.589735	0.628111	0.534411	0.542993	0.560256
HC17	0.605031	0.546263	0.548420	0.451290	0.430438	0.365883
HC18	0.369741	0.461153	0.495022	0.400456	0.392256	0.361063
HC19	0.500950	0.548326	0.579338	0.452100	0.422277	0.415222
HC20	0.492597	0.582005	0.649683	0.511370	0.477784	0.443537
HC21	0.463582	0.513963	0.729320	0.576859	0.521165	0.492085

HC22	0.574187	0.573689	0.675429	0.535616	0.476202	0.484148
HC23	0.622592	0.675599	0.772993	0.642631	0.515357	0.512341
HC24	0.514681	0.597712	0.710026	0.444147	0.401300	0.413179

Abbreviations: HC, healthy control; ID, patient identification number; PSI, phase synchrony index.

Supplementary table S15. left Intrah-PSI value of each patient

ID	δ	θ	α	β1	β2	γ
1	0.626658	0.568320	0.566960	0.550921	0.561148	0.527656
2	0.561583	0.677632	0.723188	0.593558	0.548761	0.521378
3	0.563540	0.549947	0.689077	0.595675	0.565095	0.516587
4	0.593724	0.645087	0.699043	0.613896	0.583774	0.577776
5	0.484598	0.499933	0.649272	0.565250	0.539222	0.503628
6	0.565550	0.575869	0.567858	0.545785	0.569714	0.640861
7	0.508621	0.538611	0.537695	0.483952	0.485107	0.500106
8	0.476133	0.517371	0.560527	0.476629	0.452446	0.448677
9	0.529933	0.567493	0.597768	0.556029	0.542141	0.529961
10	0.717309	0.630112	0.670948	0.591873	0.571304	0.570298
11	0.468740	0.538921	0.625012	0.557151	0.520798	0.500526
12	0.519943	0.513091	0.575946	0.521268	0.477521	0.472150
13	0.519617	0.546718	0.604821	0.510465	0.477489	0.481765
14	0.499429	0.554858	0.598228	0.571891	0.540136	0.539376
15	0.508810	0.577379	0.640635	0.566659	0.551619	0.548554
16	0.541315	0.574907	0.622060	0.565751	0.558430	0.559991
17	0.487323	0.527029	0.626117	0.580437	0.562499	0.578401
18	0.578763	0.537917	0.537074	0.448113	0.471376	0.495297
19	0.502951	0.505758	0.616298	0.548659	0.512799	0.474363
20	0.549628	0.615575	0.661777	0.539569	0.489063	0.505956
21	0.578000	0.610692	0.689286	0.562452	0.527662	0.503345

22	0.539649	0.543952	0.584333	0.558237	0.477145	0.481595
23	0.583755	0.565194	0.636294	0.538547	0.539477	0.499401
24	0.482864	0.506978	0.517557	0.499649	0.485954	0.464596
25	0.505761	0.507802	0.562536	0.525119	0.540533	0.574051
26	0.495348	0.551062	0.540736	0.464019	0.474691	0.510391
27	0.555929	0.539872	0.581324	0.478902	0.470248	0.486336
28	0.549819	0.565266	0.590435	0.584335	0.603288	0.629139
29	0.540645	0.574879	0.654405	0.595566	0.577254	0.532698
30	0.477543	0.501512	0.549303	0.518461	0.521035	0.524720
31	0.541219	0.663040	0.697320	0.519862	0.531236	0.547493

Abbreviations: ID, patient identification number; Intrah, intrahemispheric; PSI, phase synchrony index.

Supplementary table S16. right Intrah-PSI value of each patient

ID	δ	θ	α	β1	β2	γ
1	0.656302	0.661197	0.742329	0.578232	0.530959	0.505573
2	0.672036	0.691448	0.704095	0.573124	0.523921	0.487592
3	0.655719	0.669237	0.809491	0.633927	0.566664	0.534649
4	0.628449	0.707095	0.765181	0.650855	0.633158	0.656505
5	0.548657	0.583191	0.745006	0.612739	0.561153	0.514914
6	0.541234	0.554506	0.521590	0.478617	0.498832	0.559621
7	0.649438	0.634321	0.601950	0.514479	0.518420	0.561145
8	0.594959	0.613661	0.653141	0.555363	0.510772	0.507603
9	0.610576	0.626761	0.619638	0.563099	0.547919	0.549824
10	0.748725	0.649971	0.647223	0.555415	0.553266	0.598668
11	0.586488	0.542253	0.645845	0.495312	0.454702	0.427521
12	0.633617	0.607095	0.646491	0.588563	0.526176	0.510360
13	0.609862	0.584597	0.614325	0.499241	0.480558	0.481717
14	0.552988	0.601866	0.661717	0.600914	0.533377	0.518052
15	0.707012	0.714107	0.727523	0.572472	0.510981	0.501335
16	0.604567	0.636047	0.628005	0.549719	0.553412	0.579384
17	0.705092	0.650995	0.621609	0.547351	0.546958	0.575343
18	0.549581	0.518464	0.583718	0.512902	0.557640	0.664217
19	0.692670	0.638129	0.702756	0.530317	0.485308	0.453251
20	0.599691	0.670595	0.707555	0.595526	0.555913	0.583223
21	0.582671	0.627619	0.697525	0.538145	0.503480	0.494505

22	0.690989	0.710922	0.700971	0.639141	0.568593	0.562436
23	0.602016	0.628160	0.756362	0.587980	0.542098	0.502460
24	0.627572	0.608757	0.592762	0.491719	0.463684	0.429366
25	0.517756	0.530572	0.575458	0.524933	0.523020	0.555221
26	0.603195	0.537099	0.502519	0.446466	0.443187	0.472841
27	0.672679	0.656296	0.636215	0.500012	0.497222	0.528662
28	0.558434	0.617899	0.630005	0.527107	0.529798	0.559845
29	0.503623	0.545827	0.679138	0.647970	0.621321	0.579422
30	0.565406	0.637750	0.637546	0.527676	0.500283	0.515981
31	0.570548	0.644019	0.632156	0.503115	0.512436	0.521985

Abbreviations: ID, patient identification number; Intrah, intrahemispheric; PSI, phase synchrony index.

Supplementary table S17. left Intrah-PSI value of each healthy control participant

ID	δ	θ	α	β1	β2	γ
HC1	0.549167	0.605464	0.716657	0.597495	0.576755	0.538985
HC2	0.604503	0.632756	0.701726	0.616429	0.570574	0.556965
HC3	0.475154	0.474206	0.563222	0.513304	0.509562	0.529704
HC4	0.569090	0.621328	0.623776	0.579461	0.548004	0.526079
HC5	0.552802	0.562253	0.585100	0.566415	0.551724	0.567856
HC6	0.445399	0.520553	0.606805	0.563180	0.532012	0.529537
HC7	0.593697	0.549261	0.578332	0.545064	0.543569	0.521994
HC8	0.466057	0.564309	0.685614	0.528777	0.498783	0.485324
HC9	0.530928	0.553089	0.570569	0.535884	0.520888	0.470998
HC10	0.597427	0.617489	0.729532	0.616868	0.541076	0.531398
HC11	0.598202	0.565343	0.618975	0.559658	0.549647	0.502332
HC12	0.513358	0.549627	0.672173	0.513350	0.480795	0.445143
HC13	0.587306	0.547290	0.560092	0.572776	0.602525	0.650433
HC14	0.571371	0.637619	0.693566	0.608693	0.546472	0.468773
HC15	0.532380	0.547712	0.683856	0.501794	0.473833	0.461426
HC16	0.480812	0.590504	0.653048	0.588989	0.580065	0.582767
HC17	0.535555	0.542659	0.564525	0.490292	0.477492	0.422837
HC18	0.453364	0.500228	0.607166	0.508070	0.496680	0.459050
HC19	0.513927	0.545903	0.560381	0.547233	0.553124	0.540858
HC20	0.489905	0.549231	0.655674	0.556250	0.543056	0.527247
HC21	0.458042	0.504676	0.617511	0.549285	0.550461	0.530716

HC22	0.527757	0.567669	0.666524	0.564234	0.538868	0.513393
HC23	0.559159	0.581542	0.676380	0.558403	0.485421	0.453035
HC24	0.521156	0.598772	0.716512	0.534420	0.490402	0.445460

Abbreviations: HC, healthy control; ID, patient identification number; Intrah, intrahemispheric; PSI, phase synchrony index.

Supplementary table S18. right Intrah-PSI value of each healthy control participant

ID	δ	θ	α	β1	β2	γ
HC1	0.635094	0.687104	0.768674	0.621075	0.574418	0.546385
HC2	0.559977	0.594879	0.672883	0.602246	0.572849	0.552207
HC3	0.567133	0.540809	0.630751	0.526253	0.514350	0.520457
HC4	0.572057	0.587861	0.577505	0.546561	0.533574	0.517223
HC5	0.568223	0.584800	0.585046	0.571279	0.564258	0.567794
HC6	0.458067	0.510533	0.596383	0.540207	0.519952	0.504920
HC7	0.600802	0.533158	0.539449	0.505125	0.503682	0.460230
HC8	0.471386	0.570106	0.700734	0.545197	0.509421	0.492012
HC9	0.485849	0.513662	0.567777	0.531697	0.504507	0.446609
HC10	0.576964	0.601064	0.712230	0.607058	0.538529	0.538624
HC11	0.613923	0.577412	0.591000	0.570939	0.548979	0.513614
HC12	0.530428	0.554066	0.686745	0.531813	0.491985	0.463797
HC13	0.594094	0.574432	0.574883	0.588732	0.611016	0.641659
HC14	0.600475	0.639558	0.687401	0.608661	0.556013	0.536832
HC15	0.556682	0.572337	0.710873	0.553744	0.514536	0.502749
HC16	0.483330	0.611770	0.663599	0.586721	0.584257	0.585467
HC17	0.651910	0.590871	0.613287	0.528679	0.515541	0.456670
HC18	0.477874	0.517223	0.599463	0.508200	0.488285	0.444027
HC19	0.528225	0.560708	0.589606	0.515279	0.508999	0.504251
HC20	0.498710	0.565773	0.648501	0.557097	0.537294	0.487841
HC21	0.493616	0.532037	0.699847	0.585523	0.567313	0.531292

HC22	0.483996	0.531564	0.635902	0.526768	0.518017	0.481829
HC23	0.584382	0.579421	0.676790	0.547077	0.466590	0.441116
HC24	0.526848	0.609719	0.739196	0.534372	0.476426	0.430800

Abbreviations: HC, healthy control; ID, patient identification number; Intrah, intrahemispheric; PSI, phase synchrony index.

Supplementary table S19. F7F8-PLI value of each patient

ID	δ	θ	α	β1	β2	γ
1	0.302785	0.320633	0.379863	0.289590	0.315358	0.291749
2	0.368736	0.311284	0.372015	0.275653	0.294471	0.301629
3	0.346422	0.307880	0.436806	0.303667	0.311399	0.293119
4	0.278740	0.299264	0.369389	0.283051	0.288072	0.299048
5	0.297641	0.291706	0.411598	0.300635	0.307535	0.289001
6	0.288742	0.288500	0.302495	0.302379	0.287863	0.304759
7	0.295780	0.313070	0.311443	0.286750	0.288744	0.303052
8	0.269590	0.307781	0.318940	0.301165	0.312724	0.299582
9	0.306368	0.330709	0.343653	0.286689	0.313912	0.329608
10	0.286181	0.293991	0.340304	0.287404	0.298486	0.296945
11	0.275429	0.299364	0.445847	0.307141	0.314107	0.293150
12	0.306727	0.298964	0.350983	0.305437	0.296122	0.296125
13	0.314343	0.362272	0.428801	0.297592	0.288048	0.296830
14	0.344339	0.299610	0.332857	0.293837	0.303605	0.285112
15	0.337285	0.317750	0.323877	0.293411	0.293004	0.290151
16	0.294709	0.304445	0.348080	0.294356	0.290589	0.289818
17	0.277784	0.288755	0.317074	0.291037	0.295202	0.284170
18	0.328885	0.290390	0.311618	0.291869	0.309634	0.309513
19	0.296638	0.314164	0.389197	0.290883	0.288549	0.282153
20	0.337104	0.277671	0.332739	0.264087	0.281991	0.314451
21	0.261307	0.269759	0.349452	0.276405	0.329484	0.344004

22	0.292776	0.323422	0.340521	0.309396	0.270895	0.291386
23	0.275102	0.304999	0.509548	0.291584	0.298541	0.301744
24	0.306659	0.307618	0.312324	0.297528	0.304077	0.281658
25	0.295075	0.309222	0.331134	0.299410	0.301527	0.298259
26	0.281772	0.328431	0.298782	0.301913	0.292525	0.305322
27	0.317937	0.279285	0.298486	0.292504	0.304335	0.305830
28	0.294684	0.321304	0.342657	0.294883	0.310973	0.295271
29	0.299754	0.298130	0.316956	0.292866	0.308279	0.294565
30	0.279528	0.292707	0.332537	0.279500	0.300472	0.288298
31	0.336700	0.409954	0.450788	0.270115	0.313454	0.307891

Abbreviations: ID, patient identification number; PLI, phase lag index.

Supplementary table S20. F7T5-PLI value of each patient

ID	δ	θ	α	β1	β2	γ
1	0.284153	0.288906	0.358622	0.278501	0.305150	0.287174
2	0.271391	0.302573	0.363555	0.290758	0.283718	0.297792
3	0.289162	0.307437	0.525397	0.349489	0.314194	0.293734
4	0.301446	0.329237	0.423928	0.302734	0.288913	0.299438
5	0.316280	0.288775	0.423370	0.316227	0.308492	0.286705
6	0.272156	0.294974	0.312497	0.293406	0.281146	0.300601
7	0.321774	0.321660	0.334538	0.294013	0.297772	0.299409
8	0.282726	0.298164	0.364550	0.291238	0.296345	0.292075
9	0.337730	0.321947	0.348458	0.292439	0.286652	0.286829
10	0.315907	0.278307	0.433207	0.292521	0.284757	0.304440
11	0.330704	0.386183	0.389961	0.277537	0.288191	0.294930
12	0.252037	0.278097	0.363676	0.322787	0.297335	0.295182
13	0.314755	0.331305	0.444025	0.286721	0.291640	0.304893
14	0.277177	0.299566	0.367319	0.337909	0.296605	0.288564
15	0.270748	0.328243	0.403133	0.281699	0.296693	0.292428
16	0.308564	0.353106	0.388060	0.286768	0.291734	0.286830
17	0.283570	0.284868	0.330430	0.291167	0.292614	0.298669
18	0.317611	0.286513	0.372379	0.276559	0.293315	0.299234
19	0.318047	0.296203	0.419199	0.316366	0.289531	0.285922
20	0.331198	0.258312	0.441794	0.361955	0.292672	0.317345
21	0.277642	0.301879	0.471179	0.295789	0.295283	0.304736

22	0.284947	0.306538	0.329225	0.304711	0.273243	0.295352
23	0.305430	0.304028	0.438040	0.283665	0.303103	0.281191
24	0.277180	0.295488	0.333414	0.305937	0.299942	0.284541
25	0.307855	0.301535	0.399475	0.286591	0.292393	0.289613
26	0.284928	0.298832	0.304260	0.287789	0.297530	0.306419
27	0.304003	0.286081	0.332197	0.283234	0.299661	0.302538
28	0.276744	0.299795	0.360069	0.279823	0.297497	0.302440
29	0.283513	0.305369	0.406759	0.323201	0.322529	0.296846
30	0.252448	0.303675	0.320076	0.280757	0.289803	0.293494
31	0.269012	0.493916	0.567896	0.269825	0.302200	0.302517

Abbreviations: ID, patient identification number; PLI, phase lag index.

Supplementary table S21. F8T6-PLI value of each patient

ID	δ	θ	α	β1	β2	γ
1	0.297278	0.270365	0.348074	0.286078	0.311913	0.285899
2	0.279035	0.392421	0.560089	0.330512	0.297839	0.283096
3	0.307294	0.326366	0.589786	0.338553	0.313203	0.280965
4	0.286131	0.369169	0.542895	0.360732	0.299159	0.301539
5	0.296655	0.301289	0.526494	0.350912	0.310385	0.287115
6	0.291825	0.315574	0.316134	0.307354	0.287419	0.303639
7	0.297439	0.331118	0.369876	0.302224	0.303186	0.286481
8	0.271751	0.332633	0.358464	0.329279	0.306128	0.297013
9	0.345872	0.342343	0.376400	0.294110	0.298957	0.297073
10	0.325697	0.297222	0.455188	0.283764	0.299873	0.300168
11	0.307868	0.351355	0.551904	0.330264	0.308005	0.290921
12	0.277122	0.297016	0.444802	0.371454	0.305895	0.299733
13	0.268259	0.371827	0.471525	0.291115	0.282081	0.291019
14	0.311368	0.308210	0.494314	0.427543	0.317153	0.287134
15	0.302625	0.295708	0.396712	0.298539	0.287076	0.295919
16	0.294406	0.328416	0.405598	0.287099	0.294448	0.287939
17	0.270006	0.291149	0.365233	0.307733	0.290125	0.295516
18	0.313965	0.281472	0.390240	0.313689	0.293878	0.291144
19	0.302984	0.323012	0.616469	0.353253	0.308056	0.289899
20	0.365894	0.285865	0.450111	0.346179	0.286636	0.305588
21	0.256341	0.325486	0.594418	0.341903	0.292089	0.299689

22	0.296844	0.325165	0.403184	0.407445	0.319156	0.295906
23	0.316048	0.351764	0.660133	0.360572	0.302865	0.290097
24	0.308128	0.293508	0.341409	0.295543	0.303587	0.285430
25	0.283970	0.308963	0.412237	0.315825	0.315805	0.300412
26	0.310588	0.332981	0.334108	0.286367	0.283101	0.304950
27	0.313853	0.300432	0.325561	0.310785	0.311689	0.320458
28	0.297822	0.333374	0.420131	0.311222	0.302477	0.296594
29	0.290772	0.280489	0.498173	0.386966	0.334491	0.289869
30	0.287531	0.319285	0.411126	0.305099	0.304093	0.289969
31	0.309848	0.333139	0.364516	0.265551	0.287810	0.302564

Abbreviations: ID, patient identification number; PLI, phase lag index.

Supplementary table S22. F7F8-PLI value of each healthy control participant

ID	δ	θ	α	β1	β2	γ
HC1	0.297364	0.286724	0.338286	0.262241	0.290985	0.293724
HC2	0.287660	0.309746	0.326720	0.301481	0.304396	0.299521
HC3	0.313421	0.276605	0.336440	0.290343	0.293817	0.307761
HC4	0.274768	0.296678	0.324985	0.305601	0.327120	0.294583
HC5	0.283027	0.298071	0.307215	0.296929	0.324529	0.322938
HC6	0.303336	0.303145	0.317043	0.302751	0.316708	0.321281
HC7	0.311813	0.272640	0.316846	0.300077	0.309494	0.294497
HC8	0.314130	0.288999	0.378985	0.311538	0.324492	0.291695
HC9	0.301128	0.274115	0.346598	0.313194	0.314022	0.283255
HC10	0.275787	0.285604	0.411714	0.313754	0.310746	0.328600
HC11	0.289458	0.277312	0.330155	0.292514	0.296580	0.311045
HC12	0.285463	0.288083	0.358866	0.283003	0.300905	0.282552
HC13	0.273415	0.282866	0.298478	0.306775	0.301875	0.307022
HC14	0.285473	0.304082	0.346281	0.296971	0.294118	0.298873
HC15	0.261911	0.274232	0.349026	0.265838	0.300767	0.306524
HC16	0.263943	0.302138	0.349010	0.296270	0.307777	0.287261
HC17	0.279009	0.270962	0.324295	0.293240	0.320361	0.310086
HC18	0.283247	0.288847	0.330153	0.280133	0.319167	0.287814
HC19	0.283902	0.284449	0.308113	0.298890	0.306035	0.298009
HC20	0.273693	0.294666	0.336459	0.282625	0.307484	0.303409
HC21	0.310111	0.304336	0.339077	0.286903	0.301965	0.288742

HC22	0.278051	0.278764	0.334053	0.272332	0.317822	0.308883
HC23	0.266226	0.284861	0.319445	0.267581	0.308910	0.307021
HC24	0.294801	0.311950	0.353548	0.280705	0.310054	0.285480

Abbreviations: HC, healthy control; ID, patient identification number; PLI, phase lag index.

Supplementary table S23. F7T5-PLI value of each healthy control participant

ID	δ	θ	α	β1	β2	γ
HC1	0.265967	0.342733	0.509152	0.297776	0.320056	0.290880
HC2	0.314413	0.306926	0.441756	0.307720	0.303648	0.292190
HC3	0.287894	0.299702	0.351124	0.284289	0.288230	0.300529
HC4	0.293939	0.299941	0.305599	0.296415	0.313626	0.289647
HC5	0.296662	0.297645	0.304751	0.291802	0.310893	0.299891
HC6	0.287672	0.305787	0.368144	0.314046	0.311109	0.317859
HC7	0.329988	0.297728	0.356743	0.307882	0.337793	0.295998
HC8	0.317506	0.347769	0.532065	0.362458	0.309441	0.299659
HC9	0.292841	0.293131	0.344434	0.335002	0.341062	0.289847
HC10	0.303098	0.285211	0.493697	0.365918	0.293883	0.305115
HC11	0.289744	0.300939	0.399862	0.327281	0.354135	0.309993
HC12	0.272080	0.308543	0.513212	0.304539	0.299224	0.277706
HC13	0.288258	0.320572	0.326222	0.300746	0.282274	0.305675
HC14	0.303260	0.317144	0.407002	0.317544	0.305005	0.286471
HC15	0.293027	0.286731	0.474918	0.323071	0.296616	0.293702
HC16	0.311669	0.268368	0.367605	0.284145	0.300437	0.285718
HC17	0.280623	0.304148	0.333652	0.297077	0.316435	0.288809
HC18	0.269496	0.301361	0.381859	0.289792	0.315834	0.293950
HC19	0.325300	0.294625	0.312222	0.312149	0.310730	0.297928
HC20	0.279564	0.293343	0.394693	0.323539	0.330437	0.286966
HC21	0.292363	0.287640	0.361781	0.291667	0.310422	0.287678

HC22	0.305087	0.296815	0.402217	0.312103	0.315221	0.298120
HC23	0.311469	0.287977	0.427038	0.359102	0.318548	0.298945
HC24	0.310237	0.346210	0.564006	0.318710	0.311172	0.284754

Abbreviations: HC, healthy control; ID, patient identification number; PLI, phase lag index.

Supplementary table S24. F8T6-PLI value of each healthy control participant

ID	δ	θ	α	β1	β2	γ
HC1	0.299508	0.353843	0.569286	0.332578	0.314176	0.291530
HC2	0.271136	0.309730	0.454929	0.309477	0.299942	0.286664
HC3	0.285827	0.308337	0.424629	0.321636	0.299544	0.308380
HC4	0.304892	0.286045	0.306603	0.316663	0.323714	0.286803
HC5	0.306643	0.284245	0.303760	0.312083	0.312519	0.295965
HC6	0.324151	0.280095	0.363853	0.297355	0.299301	0.293879
HC7	0.282133	0.295230	0.358178	0.320099	0.326980	0.281409
HC8	0.319306	0.393121	0.591757	0.369803	0.307820	0.296433
HC9	0.293212	0.294572	0.358519	0.331469	0.317668	0.291928
HC10	0.288582	0.327316	0.542781	0.371938	0.301753	0.304580
HC11	0.301845	0.290730	0.331037	0.292375	0.328631	0.287839
HC12	0.266556	0.325575	0.602447	0.345465	0.296627	0.271261
HC13	0.240910	0.296467	0.305497	0.308646	0.285579	0.299153
HC14	0.300866	0.294015	0.473935	0.343080	0.304276	0.280202
HC15	0.272952	0.331564	0.512119	0.308015	0.309150	0.297346
HC16	0.298211	0.294866	0.345254	0.289007	0.300822	0.280912
HC17	0.269312	0.316762	0.410513	0.330645	0.326888	0.297020
HC18	0.270333	0.289743	0.364214	0.293070	0.321646	0.287486
HC19	0.311362	0.290714	0.305919	0.301508	0.303155	0.297195
HC20	0.287214	0.312389	0.427152	0.334512	0.327612	0.291181
HC21	0.264297	0.293149	0.537025	0.327764	0.311991	0.284184

HC22	0.307907	0.285403	0.399856	0.304221	0.316293	0.291148
HC23	0.275790	0.298881	0.461555	0.352584	0.315003	0.298505
HC24	0.311634	0.419424	0.644855	0.329014	0.301242	0.284333

Abbreviations: HC, healthy control; ID, patient identification number; PLI, phase lag index.

Supplementary table S25. F7F8-wP value of each patient

ID	δ	θ	α	β1	β2	γ
1	-0.081561	-0.412769	-2.765739	-0.748973	-0.302316	0.284228
2	0.718974	-0.010679	-0.446364	-0.098688	0.011275	0.099063
3	0.276895	0.782759	1.148601	-0.197110	0.275584	1.004526
4	0.996382	0.011373	-0.361869	-0.077810	0.004615	0.070438
5	-1.175396	-0.494225	-2.501502	-0.663271	-0.344329	-0.191987
6	-4.011456	0.144912	-3.097891	-16.825480	-26.563731	-57.452182
7	-0.490966	0.870525	1.105530	0.650854	0.671519	1.169161
8	-1.431229	-1.260979	-6.476913	-4.878357	-3.105219	-0.770964
9	-1.728326	-1.671893	-1.971610	-0.473182	-0.586012	-0.355468
10	-4.787969	-1.180478	-0.090868	-0.209704	-0.114809	-0.023853
11	3.797087	-0.248690	-3.030989	-2.049381	-1.834332	-1.190941
12	2.100951	0.519212	0.346494	0.104398	0.053082	0.174733
13	1.012505	1.025442	0.706822	0.086197	0.050751	0.077124
14	1.918772	-0.573892	-2.095996	-1.935815	-0.848406	0.054050
15	1.249122	0.242649	-0.233473	-0.346165	-0.274988	-0.187337
16	5.370255	-0.394903	-2.327214	-0.486097	-0.140525	0.029507
17	1.478058	0.403106	-1.232474	-0.780764	-0.619331	-0.324262
18	1.838131	0.098346	0.143652	1.645758	5.053350	10.171409
19	4.137082	0.445078	-1.006644	-1.059788	-0.542686	-0.099403
20	1.774197	-0.567207	-1.160893	0.400205	1.012197	1.380535
21	-0.949650	-0.126764	-1.980085	-0.362520	-0.357179	1.144770

22	1.125822	-0.135841	-0.307865	-0.139575	0.456224	1.502795
23	0.137340	-0.350513	-3.691484	0.630414	1.829356	2.858897
24	1.317017	0.109096	-1.665473	-1.000012	-1.087422	-1.312385
25	-1.560139	-0.156889	-0.456890	-0.694281	-0.890974	-0.462142
26	-3.361783	-1.103152	0.616917	4.625158	12.118428	16.615715
27	0.874799	0.428766	-0.229382	-2.206555	-4.878210	-10.042788
28	-1.976766	-0.771491	-0.951765	-0.276000	-0.235112	0.004675
29	0.707958	0.075642	-0.192309	-0.120243	0.057378	0.273987
30	10.802192	1.073542	-11.306911	-3.848575	-1.369624	0.336946
31	1.590254	0.690011	-0.010872	-0.028438	-0.065626	0.307260

Abbreviations: ID, patient identification number; wP, wavelet power.

Supplementary table S26. F7T5-wP value of each patient

ID	δ	θ	α	β1	β2	γ
1	-8.416302	0.118535	-0.118725	0.418352	0.473570	0.664571
2	2.552714	0.276661	0.154610	0.072426	0.089784	0.116239
3	3.016004	1.061417	2.124429	0.466272	0.492123	1.077011
4	2.582716	0.636140	0.313469	0.004209	0.162705	0.326195
5	2.393022	0.097074	-2.656233	-0.463538	-0.001102	0.110188
6	6.794718	-2.123981	-7.239881	0.700157	8.035344	18.597562
7	1.574543	-0.668948	0.399854	0.680281	0.799533	1.227163
8	-0.333081	-7.743890	-27.808561	-3.080922	-0.274679	-0.515173
9	-1.412045	0.945772	0.719099	-0.485397	0.114647	0.234230
10	4.886813	0.773218	-0.151823	-0.193543	0.032268	0.229707
11	1.510486	-6.248852	-2.559249	-0.444602	-0.187174	-0.124696
12	4.667122	0.787963	-0.460438	-0.432093	-0.048768	0.128806
13	-7.984434	-10.651149	-9.016040	-1.107920	-0.636794	-0.686995
14	3.019037	-0.716707	-5.550658	-3.558812	-0.791077	-0.010512
15	2.117852	0.308761	-0.591822	-0.450837	-0.161250	0.379008
16	8.024688	2.007606	-0.921029	-0.413189	-0.031393	0.161763
17	2.219479	1.127438	0.752497	0.101965	-0.018183	0.003181
18	7.129474	0.309970	-0.649435	1.141586	5.195225	10.851545
19	5.760711	1.770861	2.693350	0.519035	-0.256969	-0.632309
20	8.626452	1.582224	1.351342	1.046649	1.127488	1.495817
21	6.000239	2.610050	5.967774	-0.083849	1.053056	2.387176

22	2.727111	0.983428	0.659095	0.337505	0.903279	2.201475
23	0.272732	-4.716241	-23.937688	-1.281646	1.887700	4.006942
24	2.119375	0.369215	-0.128856	0.018380	0.011982	-0.011800
25	1.861162	0.109550	-3.935521	-0.443644	-0.041473	0.071956
26	3.648818	-2.434570	-0.928684	7.990691	17.868958	27.118462
27	4.417480	0.561564	0.075157	0.828933	1.891662	3.359672
28	0.870303	-0.342749	-0.930184	-0.143789	0.064826	0.178322
29	1.544134	0.018970	-2.119404	-0.923782	-0.018646	0.795742
30	13.601271	6.404006	-4.456517	-0.891313	1.200644	1.926859
31	2.903787	1.944607	1.897180	0.858365	2.199844	4.295970

Abbreviations: ID, patient identification number; wP, wavelet power.

Supplementary table S27. F8T6-wP value of each patient

ID	δ	θ	α	β1	β2	γ
1	0.731876	0.490084	0.093712	0.130441	0.283210	0.244382
2	2.452822	0.135436	-1.615842	-0.383182	-0.073655	-0.001035
3	3.028993	0.826925	1.738035	-0.121672	-0.035894	0.168220
4	1.650168	0.615575	-0.099640	-0.008210	0.136976	0.274485
5	4.243107	0.948274	1.118540	0.442218	0.445884	0.376147
6	11.041033	-1.211991	-3.070328	16.405937	33.437294	74.707910
7	3.520119	0.708159	-0.939258	-0.291105	0.059849	0.099265
8	1.170637	-1.263983	-0.837545	-4.094267	-0.660362	-0.254559
9	2.039724	1.481180	0.240082	-0.443097	0.498105	0.561002
10	9.473201	2.321482	0.090054	-0.376014	-0.094183	0.153623
11	1.290200	-1.278528	-7.626862	-0.335215	1.071571	0.957321
12	2.705713	0.537808	-0.379959	-0.361468	0.007326	0.046521
13	1.057384	-12.941044	-17.352400	-1.135867	-0.278171	-0.177167
14	1.500681	0.501934	-6.286889	-2.460750	-0.028379	-0.116181
15	1.012385	-0.239271	-2.581261	-0.491541	0.225607	0.571522
16	2.586842	2.021670	-2.349042	-0.575710	-0.247338	-0.233038
17	1.164715	0.459021	0.373019	-0.055196	-0.021366	-0.244854
18	4.281955	0.322146	-1.332404	-0.903040	0.070830	0.756517
19	-0.412436	-0.648128	-7.628816	-0.011677	-0.056092	-0.832507
20	6.540241	1.977290	2.819873	0.562437	-0.078133	-0.317047
21	6.753491	0.143679	-9.246693	-2.083491	0.928674	1.133823

22	1.423740	1.318527	0.875481	0.145874	0.248736	0.517042
23	0.369419	-1.575441	-14.856910	-1.978123	0.041057	0.964538
24	1.482820	0.461317	0.606199	0.294043	0.494913	0.773087
25	2.563997	0.258049	-2.075479	-0.304673	0.404105	0.532000
26	8.445891	-3.952946	-3.489126	1.979274	4.441963	8.363447
27	2.409606	0.215121	-0.607953	-1.527529	-1.225529	-2.829199
28	3.180217	0.254766	-1.049949	-0.825427	-0.156738	0.094330
29	0.893913	0.162965	-1.330476	-0.658254	-0.121244	0.512448
30	7.031463	6.993772	6.954869	3.205603	2.688395	1.958111
31	1.840062	5.097777	4.166936	0.768098	1.975942	3.464357

Abbreviations: ID, patient identification number; wP, wavelet power.

Supplementary table S28. F7F8-wP value of each healthy control participant

ID	δ	θ	α	β1	β2	γ
HC1	-2.933060	-1.425797	-2.070019	-0.411421	-0.260692	-0.320158
HC2	1.245988	0.044013	0.267988	0.097837	0.258143	0.416530
HC3	1.345528	0.122601	-0.110837	0.071910	0.154448	0.142556
HC4	1.621478	0.609027	0.138818	-0.364242	-0.589044	-0.133796
HC5	0.685257	-0.157967	-0.044464	-0.202759	-0.373945	-0.417946
HC6	3.599718	0.743322	0.487870	0.034723	0.119850	0.208232
HC7	-0.066326	0.081376	-0.000406	0.015724	0.051972	-0.134116
HC8	2.153719	-0.186620	-4.218064	-0.007035	0.298476	0.529680
HC9	1.023988	0.030149	-0.131626	1.058423	0.701854	-0.177696
HC10	-0.535652	0.235956	1.336574	-0.228937	-0.221244	-0.243269
HC11	-1.086737	-0.098914	0.319868	-0.018286	-0.018144	0.146500
HC12	-3.695183	-1.263939	-5.297949	-0.818500	-0.414724	-0.200107
HC13	3.771987	0.254858	0.107736	0.105878	0.174249	0.313428
HC14	-0.341395	-0.684181	-2.882825	-1.106282	-1.050846	-1.316570
HC15	-0.310192	-0.322472	-1.784994	-0.346851	-0.202868	-0.121370
HC16	0.619268	-0.235971	0.082068	-0.369315	-0.641330	-0.952102
HC17	0.130780	0.322352	0.115529	0.423352	0.157012	0.359402
HC18	2.453205	0.873428	0.998740	0.020193	-0.218585	0.015464
HC19	-0.704175	-0.202220	-0.043016	0.053214	0.100418	0.194905
HC20	0.400271	-0.011223	0.235606	0.015754	-0.006561	0.003957
HC21	-1.616149	0.518731	0.386705	0.173110	0.161697	0.333001

HC22	-2.198293	0.022977	2.302882	0.616256	0.286129	0.267158
HC23	0.373997	-0.230300	-2.834820	-2.114779	-3.353369	-3.899541
HC24	2.035269	2.004237	-2.931645	-0.355719	-0.426601	-0.618508

Abbreviations: HC, healthy control; ID, patient identification number; wP, wavelet power.

Supplementary table S29. F7T5-wP value of each healthy control participant

ID	δ	θ	α	β1	β2	γ
HC1	1.675318	-0.920583	-3.055347	-2.136528	-1.512439	-0.617955
HC2	4.089147	0.249013	-1.760723	-0.478012	-0.045422	0.596517
HC3	3.670199	0.049034	-0.674936	-0.002602	0.016589	-0.019683
HC4	1.764581	0.255016	-0.521312	-0.959163	-0.065766	0.030906
HC5	3.778266	0.599441	0.357624	0.395396	1.007259	0.899476
HC6	7.161100	0.607464	-3.392020	-1.867975	-0.735865	1.552767
HC7	2.904091	0.924167	0.710321	0.243853	0.069756	0.283126
HC8	3.285296	-2.382536	-10.704192	-0.803198	-0.165943	-0.497278
HC9	5.652523	0.488798	0.017977	-0.546568	0.067365	0.055108
HC10	10.738746	0.964913	7.744323	-3.484935	-1.730744	2.366112
HC11	3.955761	0.383332	-3.504642	-3.026159	-3.329217	-2.850236
HC12	3.737503	-0.032050	-4.426970	-0.383627	-0.199571	-0.397056
HC13	5.655229	0.410192	0.119929	0.026492	0.355318	0.867643
HC14	2.166047	0.119789	-9.988880	-5.344822	-1.305730	0.127790
HC15	0.997334	-0.138221	-1.027254	-0.889118	-0.647931	-0.624083
HC16	2.427398	-0.230910	-5.191318	-2.228568	-0.737519	-0.047709
HC17	7.945752	1.566268	0.294976	-0.050946	0.146127	0.655657
HC18	2.943710	-1.233210	-15.956082	-3.146427	-0.732306	-0.023734
HC19	0.892844	-0.000376	0.213487	0.104680	0.312939	0.630262
HC20	2.426439	0.682324	1.258901	-0.121091	-0.167645	0.121586
HC21	2.504290	0.951685	1.132144	-0.274110	0.026666	0.494309

HC22	0.681993	0.243709	1.194828	-0.497548	0.152756	0.377132
HC23	0.397259	-0.288314	-6.715397	-3.526323	-1.466541	-0.071739
HC24	3.760477	-0.906362	-11.882353	-0.799707	0.242928	0.116939

Abbreviations: ID, HC, healthy control; patient identification number; wP, wavelet power.

Supplementary table S30. F8T6-wP value of each healthy control participant

ID	δ	θ	α	β1	β2	γ
HC1	5.144454	1.242598	-2.487233	-1.244508	-0.677403	0.084146
HC2	3.005424	0.419984	-1.412029	-0.615087	-0.373608	0.076060
HC3	2.761135	0.259224	0.264903	0.123032	-0.034163	-0.110113
HC4	0.870958	0.362514	0.486622	0.588720	1.446084	0.488980
HC5	3.092680	0.758539	0.240804	0.476800	1.303036	1.270374
HC6	4.124104	-0.073793	-4.369337	-2.234962	-0.802814	1.361172
HC7	2.872515	0.804613	0.137483	0.219766	-0.320559	0.193387
HC8	0.715588	-3.954620	-14.415174	-1.208568	0.018261	0.187557
HC9	4.027072	0.360259	0.210909	-1.948088	-0.974601	0.027834
HC10	11.198483	0.355635	8.828535	-2.384564	-0.536169	2.683630
HC11	5.337451	0.896065	-1.175307	-0.708351	-0.548541	0.128601
HC12	7.078794	-0.620570	-5.128993	-0.319895	0.235230	0.091110
HC13	2.174710	0.152041	0.028358	0.003074	0.331347	0.745714
HC14	2.381367	1.149071	-3.125926	-3.221521	-0.264011	1.579500
HC15	1.295428	0.417728	1.738253	-0.206995	-0.456321	-0.903231
HC16	0.718853	0.153760	-4.187302	-1.698593	-0.129213	0.854969
HC17	8.009125	1.247610	0.154362	-0.830031	-0.461337	0.753022
HC18	0.585616	-0.958724	-11.460234	-2.753730	-0.674910	-0.228745
HC19	1.704503	0.230948	0.293421	-0.058214	-0.054124	-0.026965
HC20	2.032016	0.586603	0.615594	-0.538098	-0.282030	0.252181
HC21	3.393827	0.113236	-5.492328	-1.810845	-0.470272	0.015663

HC22	2.907402	0.148261	-1.585375	-1.616018	-0.519337	0.034079
HC23	0.018764	-0.451043	-11.185896	-5.374859	0.841246	3.318804
HC24	2.353290	-0.362287	-8.869338	-1.193749	0.513452	0.801481

Abbreviations: HC, healthy control; ID, patient identification number; wP, wavelet power.

Supplementary table S31. Comparison of the PSI values between patients and healthy control participants

Frequency band	δ		θ		α		β1		β2		γ	
Statistical value	*U*	*P*	*U*	*P*	*U*	*P*	*U*	*P*	*U*	*P*	*U*	*P*
F7F8-PSI	270.0	0.083	346.0	0.659	298.0	0.209	213.0	0.007	193.0	0.002	356.0	0.786
F7T5-PSI	338.0	0.564	283.0	0.131	328.0	0.455	342.0	0.611	260.0	0.057	221.0	0.010
F8T6-PSI	149.0	< 0.001	164.0	0.002	277.0	0.107	343.0	0.623	318.0	0.359	252.0	0.042
F3F4-PSI	329.0	0.466	318.0	0.359	278.0	0.111	239.0	0.024	227.0	0.014	333.0	0.508
F3P3-PSI	362.0	0.865	319.0	0.368	246.0	0.032	359.0	0.825	267.0	0.075	226.0	0.013
F4P4-PSI	207.0	0.005	271.0	0.087	361.0	0.852	366.0	0.919	314.0	0.325	234.0	0.019

All P values were not corrected (Mann–Whitney U test).

Abbreviation: PSI, phase synchrony index.

Supplementary table S32. Results of correlation analyses between the PSI and the ARSsp score

Frequency band	δ		θ		α		β1		β2		γ	
Statistical value	*ρ*	*P*	*ρ*	*P*	*ρ*	*P*	*ρ*	*P*	*ρ*	*P*	*ρ*	*P*
F7F8-PSI and ARSsp	-0.05	0.804	-0.13	0.485	0.29	0.109	0.55	0.001	0.21	0.267	0.12	0.510
F7T5-PSI and ARSsp	-0.05	0.774	-0.16	0.391	-0.08	0.678	-0.58	< 0.001	-0.63	< 0.001	-0.56	<0.001
F8T6-PSI and ARSsp	-0.58	< 0.001	-0.35	0.052	0.05	0.782	0.08	0.662	0.01	0.950	-0.01	0.968
F3F4-PSI and ARSsp	0.00	0.988	0.06	0.753	0.26	0.151	0.46	0.009	0.19	0.295	0.11	0.544
F3P4-PSI and ARSsp	0.12	0.520	0.03	0.889	-0.03	0.867	-0.12	0.534	-0.26	0.155	-0.25	0.180
F4P4-PSI and ARSsp	-0.22	0.242	-0.11	0.555	0.13	0.501	0.22	0.242	0.15	0.420	0.10	0.598

All P values were not corrected (Spearman’s rank correlation analysis).

Abbreviations: ARSsp, Aphasia Rating Scale speech; PSI, phase synchrony index.

Supplementary table S33. Comparison of the Intrah-PSI values between patients and healthy control participants

Frequency band	δ		θ		α		β1		β2		γ	
Statistical value	*U*	*P*	*U*	*P*	*U*	*P*	*U*	*P*	*U*	*P*	*U*	*P*
Left Intrah-PSI	362.0	0.865	340.0	0.587	280.0	0.118	325.0	0.425	339.0	0.575	322.0	0.396
Right Intrah-PSI	161.0	<0.001	165.0	<0.001	322.0	0.396	349.0	0.696	356.0	0.786	273.0	0.093

All P values were not corrected (Mann–Whitney U test).

Abbreviation: Intrah, intrahemispheric; PSI, phase synchrony index.

Supplementary table S34. Results of correlation analyses between the Intrah-PSI and the ARSsp score

Frequency band	δ		θ		α		β1		β2		γ	
Statistical value	*ρ*	*P*	*ρ*	*P*	*ρ*	*P*	*ρ*	*P*	*ρ*	*P*	*ρ*	*P*
Left Intrah-PSI and ARSsp	0.06	0.733	-0.03	0.872	0.05	0.779	-0.11	0.575	-0.23	0.209	-0.20	0.280
Right Intrah-PSI and ARSsp	-0.48	0.006	-0.22	0.239	0.20	0.277	0.39	0.028	0.35	0.051	0.22	0.238

All P values were not corrected (Spearman's rank correlation analysis).

Abbreviations: ARSsp, Aphasia Rating Scale speech; Intrah, intrahemispheric; PSI, phase synchrony index.

Supplementary table S35. Comparison of the PLI values between patients and healthy control participants

Frequency band	δ		θ		α		β1		β2		γ	
Statistical value	*U*	*P*	*U*	*P*	*U*	*P*	*U*	*P*	*U*	*P*	*U*	*P*
F7F8-PLI	237.0	0.022	160.0	<0.001	313.0	0.317	363.0	0.879	228.0	0.015	326.0	0.435
F7T5-PLI	340.0	0.587	355.0	0.773	345.0	0.647	200.0	0.004	124.0	<0.001	346.0	0.659
F8T6-PLI	291.0	0.169	268.0	0.078	334.0	0.519	358.0	0.812	228.0	0.015	302.0	0.235

All P values were not corrected (Mann–Whitney U test).

Abbreviation: PLI, phase lag index.

Supplementary table S36. Results of correlation analyses between the PLI and the ARSsp score

Frequency band	δ		θ		α		β1		β2		γ	
Statistical value	*ρ*	*P*	*ρ*	*P*	*ρ*	*P*	*ρ*	*P*	*ρ*	*P*	*ρ*	*P*
F7F8-PLI and ARSsp	-0.16	0.377	-0.37	0.038	-0.01	0.974	-0.02	0.898	0.01	0.976	0.37	0.040
F7T5-PLI and ARSsp	0.18	0.328	-0.06	0.744	0.14	0.453	0.39	0.029	0.03	0.862	-0.04	0.825
F8T6-PLI and ARSsp	0.00	0.982	0.01	0.955	0.22	0.244	0.57	<0.001	0.15	0.431	0.21	0.262

All P values were not corrected (Spearman's rank correlation analysis).

Abbreviations: ARSsp, Aphasia Rating Scale speech; PLI, phase lag index.

Supplementary table S37. Comparison of the wP values between patients and healthy control participants

Frequency band	δ		θ		α		β1		β2		γ	
Statistical value	*U*	*P*	*U*	*P*	*U*	*P*	*U*	*P*	*U*	*P*	*U*	*P*
F7F8-wP	348.0	0.684	344.0	0.635	263.0	0.064	267.0	0.075	318.0	0.359	314.0	0.325
F7T5-wP	334.0	0.519	326.0	0.435	328.0	0.455	204.0	0.004	197.0	0.003	253.0	0.043
F8T6-wP	347.0	0.671	334.0	0.519	350.0	0.709	245.0	0.031	209.0	0.006	363.0	0.879

All P values were not corrected (Mann–Whitney U test).

Abbreviation: wP, wavelet power.

Supplementary table S38. Results of correlation analyses between the wP and the ARSsp score

Frequency band	δ		θ		α		β1		β2		γ	
Statistical value	*ρ*	*P*	*ρ*	*P*	*ρ*	*P*	*ρ*	*P*	*ρ*	*P*	*ρ*	*P*
F7F8-wP and ARSsp	-0.06	0.767	-0.24	0.190	-0.26	0.159	0.11	0.569	0.13	0.487	0.29	0.114
F7T5-wP and ARSsp	0.19	0.299	0.01	0.954	-0.13	0.480	-0.21	0.258	0.22	0.233	0.31	0.092
F8T6-wP and ARSsp	0.16	0.385	0.09	0.646	-0.02	0.906	-0.15	0.429	0.11	0.561	0.12	0.540

All P values were not corrected (Spearman's rank correlation analysis).

Abbreviations: ARSsp, Aphasia Rating Scale speech; wP, wavelet power.

Supplementary table S39. Results of correlation analyses between the PSI and the LV

Frequency band	δ		θ		α		β1		β2		γ	
Statistical value	*ρ*	*P*	*ρ*	*P*	*ρ*	*P*	*ρ*	*P*	*ρ*	*P*	*ρ*	*P*
F7F8-PSI and LV	0.13	0.478	0.30	0.097	0.07	0.708	-0.23	0.207	-0.05	0.781	-0.06	0.756
F7T5-PSI and LV	-0.04	0.841	0.26	0.155	0.20	0.273	0.56	0.001	0.62	< 0.001	0.55	0.001
F8T6-PSI and LV	0.33	0.073	0.24	0.187	0.08	0.676	0.14	0.444	0.16	0.404	0.16	0.387

All P values were not corrected (Spearman’s rank correlation analysis).

Abbreviations: LV, lesion volume; PSI, phase synchrony index

Supplementary table S40. Results of correlation analyses between the PLI and the LV

Frequency band	δ		θ		α		β1		β2		γ	
Statistical value	*ρ*	*P*	*ρ*	*P*	*ρ*	*P*	*ρ*	*P*	*ρ*	*P*	*ρ*	*P*
F7F8-PLI and LV	0.23	0.214	0.38	0.034	0.31	0.093	0.02	0.933	-0.02	0.914	-0.43	0.015
F7T5-PLI and LV	-0.07	0.724	0.28	0.127	0.12	0.510	-0.14	0.438	-0.18	0.344	-0.21	0.264
F8T6-PLI and LV	0.01	0.967	0.15	0.435	0.22	0.245	-0.18	0.332	0.10	0.589	-0.35	0.051

All P values were not corrected (Spearman’s rank correlation analysis).

Abbreviations: LV, lesion volume; PLI phase lag index

Supplementary table S41. Contents of the SLTA

Field	Subscore
I. Listening	
	1 Auditory word recognition
	2 Auditory pointing by simple sentence
	3 Sequential commands
	4 Auditory pointing of syllable (kana letter)
II. Speaking	
	5 Object naming
	6 Word repetition
	7 Description of pictures
	8 Description of four panel cartoons
	9 Sentence repetition
	10 Word fluency
III. Reading	
	11 Kanji word reading
	12 Single kana letter reading
	13 Kana word reading
	14 Short sentence reading
	15 Kanji word comprehension
	16 Kana word comprehension
	17 Comprehension in written short sentence
	18 Comprehension in written command
IV. Writing	
	19 Kanji word writing
	20 Kana word writing
	21 Writing output of four panel cartoons
	22 Dictation of kana letter
	23 Dictation of kanji word
	24 Dictation of kana and kanji
	25 Dictation of short sentence
V. Calculation	
	26 Calculation

Abbreviation: SLTA, Standard Language Test of Aphasia.
